# Supplementary material for: Uterine distention as a factor in birth timing: retrospective nationwide cohort study in Sweden
Source: BMJ Open. 2018 Oct 31;8(10):e022929. doi: 10.1136/bmjopen-2018-022929 (PMC6252709; doi:10.1136/bmjopen-2018-022929)
Supplement: Supplementary file 1 [file bmjopen-2018-022929supp001.pdf]

Supplementary File

"Uterine distention as a factor in birth timing: retrospective nationwide cohort study in Sweden". Bacelis J. et al. 2018.

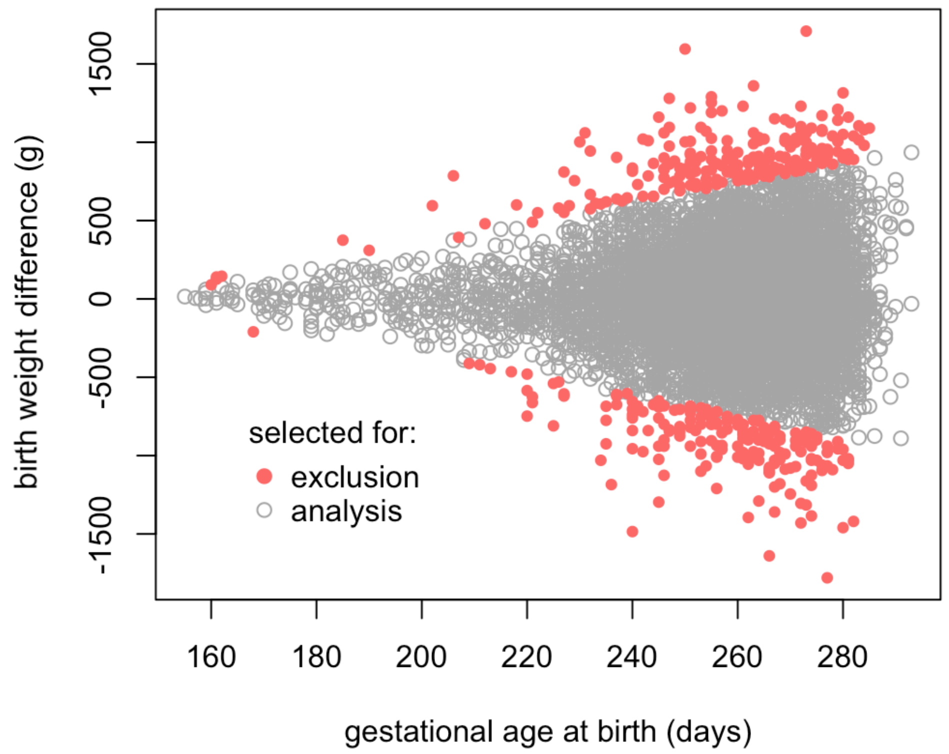

Sup Fig 1 I Selection of twin pregnancies. Pregnancies were excluded if birth weight of two twins differed by more than 2 SD of all intra-twin differences in that gestational age window. Pregnancies with thus discordant twin birth weights (n=174) were excluded from analyses.

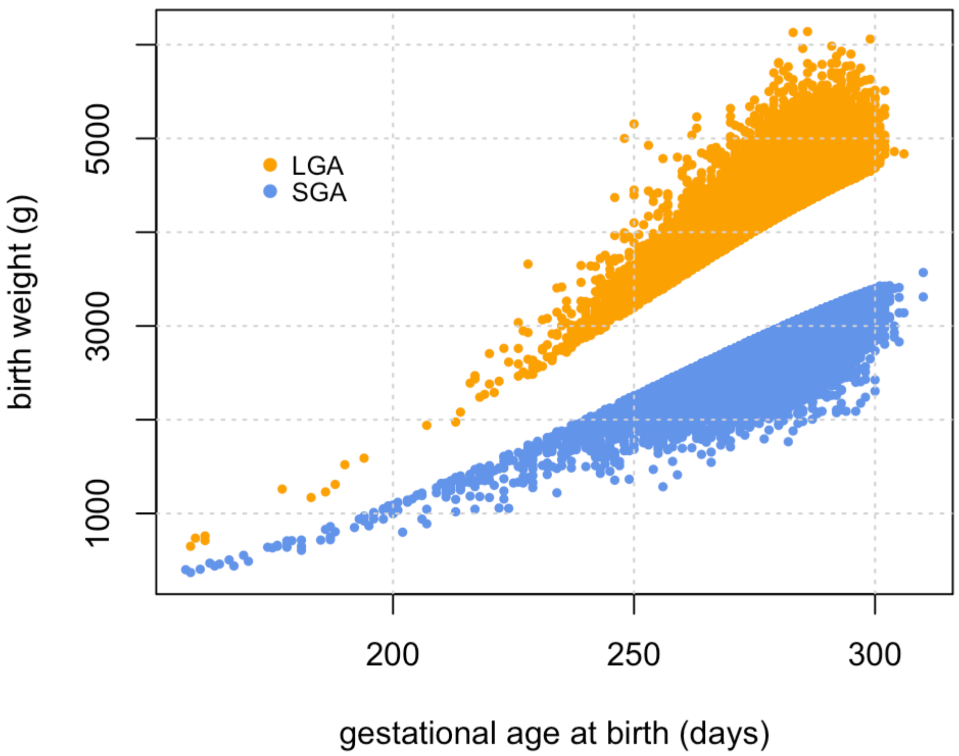

Sup Fig 2 I Classifying singleton births as small (SGA) or large (LGA) for gestational age using birth weight Z-score <-1.5 and >1.5, respectively. Birth weight Z-score was estimated from longitudinally derived intra-uterine growth curves in healthy term pregnancies using polynomial regression equation from Marsal et al.*****, which takes into account child's sex, birth weight, and ultrasound-determined gestational age at birth.

***** Marsal K, Persson PH, Larsen T, et al. Intrauterine growth curves based on ultrasonically estimated foetal weights. Acta Paediatr 1996;85(7):843-8. [published Online First: 1996/07/01].

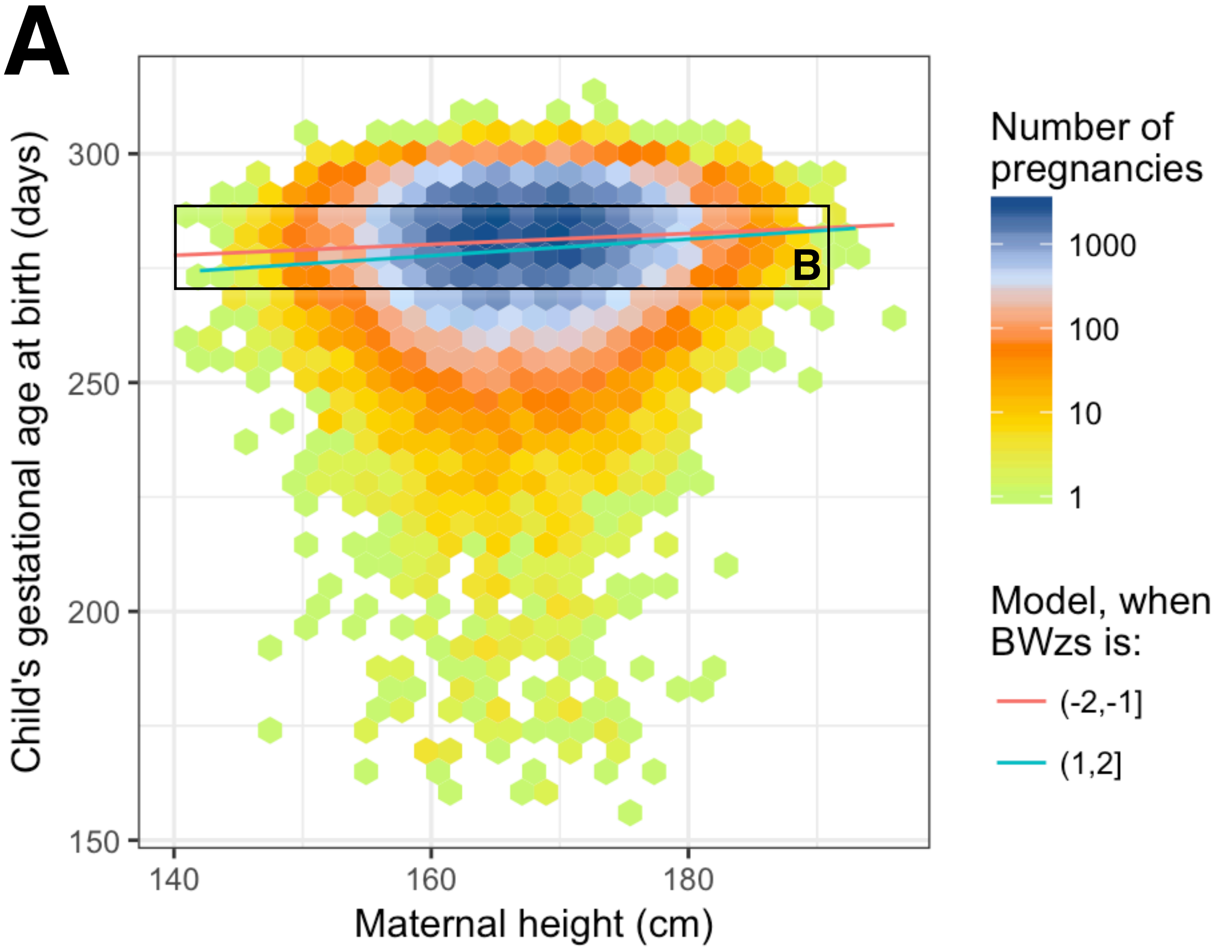

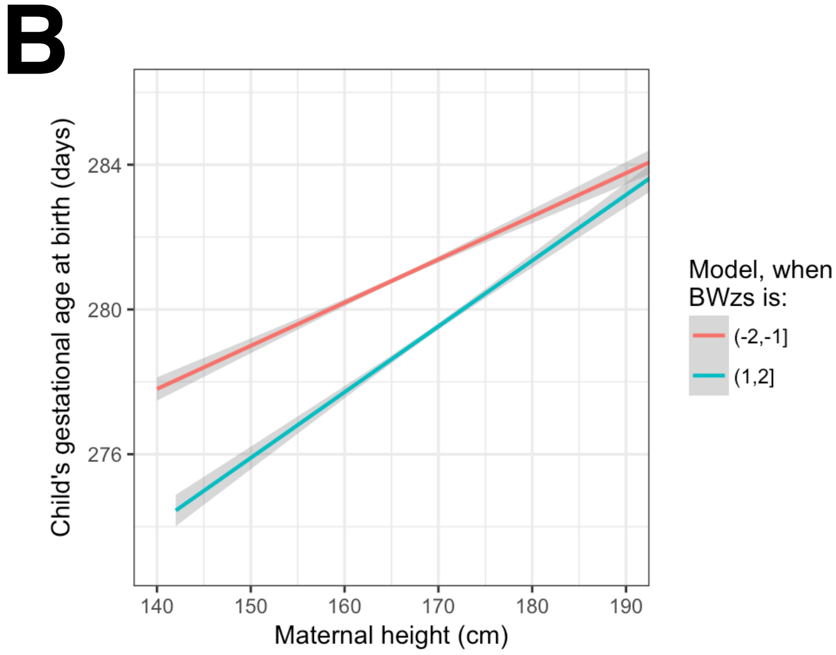

C

	Beta	Serr	Tval	Pval
**Intercept**	261,1	1,026	254,6	<1E-150
**MH**	0,119	0,006	19,3	9,4E-83
**LGA**	-12,46	1,69	-7,367	1,8E-13
**MHxLGA**	0,062	0,01	6,20	5,7E-10

Sup Fig 3 I Sensitivity analysis with different SGA and LGA definitions. SGA and LGA are defined by birth weight Z-score in ranges (-2.0, -1.0] and (1.0, 2.0], respectively. Total number of observations is 133 196 (SGA=82 471, LGA=50 725). Panel A: general view of interaction pattern; Panel B: zoomed-in view; Panel C: regression coefficients, where *MH* is maternal height and "*x*" indicates interaction. *BWzs* is birth weight Z-score.

A

	Beta	SErr	Tval	Pval
**Intercept**	253,8	0,429	592	<1E-150
**MH**	0,157	0,003	61,3	<1E-150
**BWzs**	-4,80	0,491	-9,78	1.4E-22
**MHxBWzs**	0,024	0,003	8,28	1.3E-16

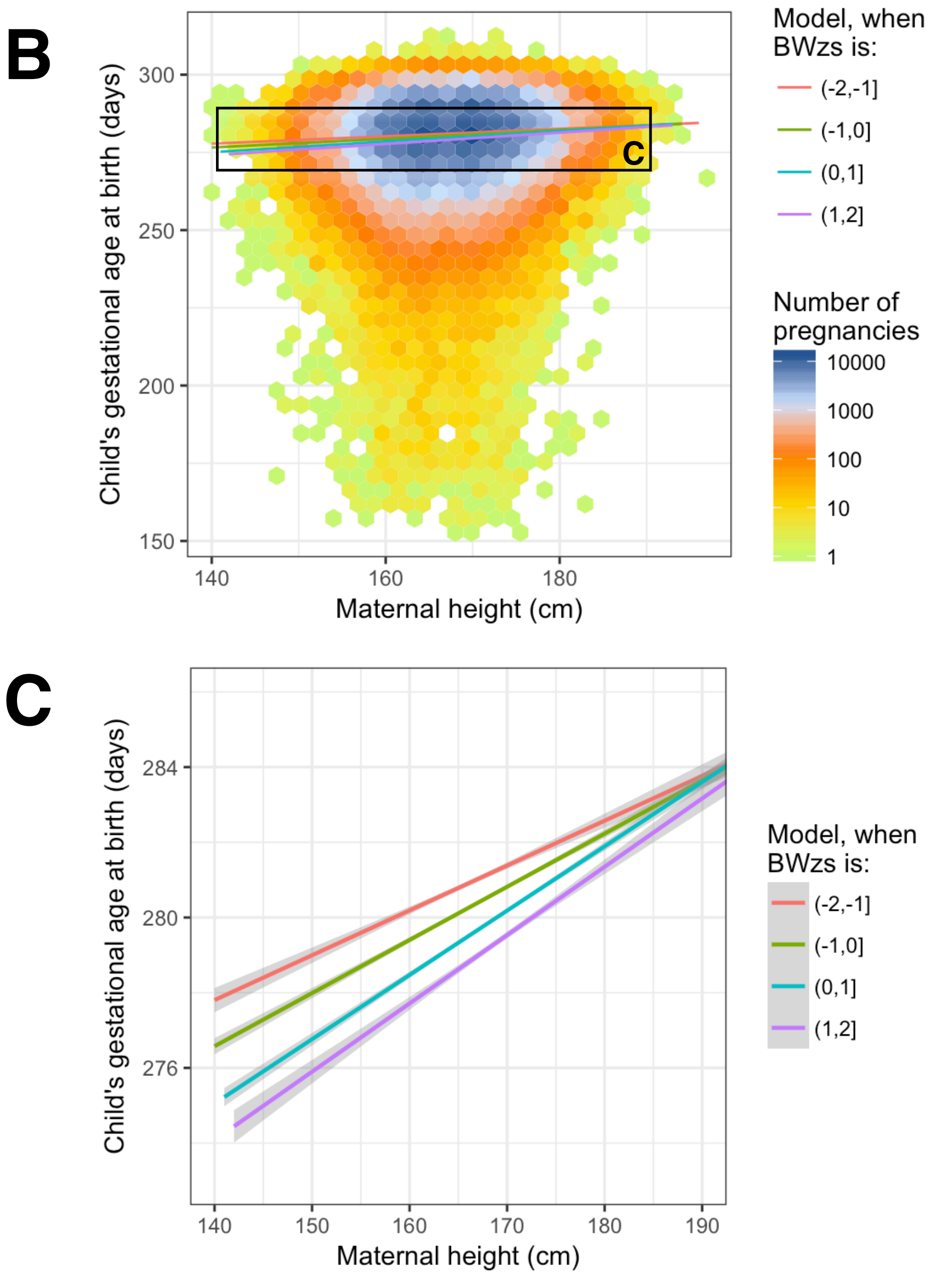

Sup Fig 4 I Sensitivity analysis with continuous uterine load variable. Only the appropriate for gestational age singletons were used, with birth weight Z-score in range (-2.0, 2.0]. In total, there were 508 442 observations. Panel A: results of regression analysis with interaction term; Panels B and C: intuitive aid for interpretation of regression coefficients (using categorized BWzs). *MH* is maternal height, *BWzs* is birth weight Z-score, and “*x*” indicates interaction.

Supplementary File

Sup Table 1 | Summary statistics of applied exclusions. This table supplements the flowchart in Figure 1, where it is equivalent to the block named "Excluded pregnancies".

order	exclusion-filter name	remaining rows	rows removed	fraction removed
1	maternal ID is missing	4185196	5304	0,13 %
2	duplicated maternal ID - child ID combination	4185184	12	0,0003 %
3	mother born (or national) outside of 4 Nordic countries	3625825	559359	13 %
4	missing, unreliable, <140 or >200 (cm) maternal height	2616038	1009787	27,8 %
5	maternal BMI <15 or >45 (kg/m^2)	2598666	17372	0,66 %
6	stillbirth	2587048	11618	0,45 %
7	missing gestational age at birth data	2584054	2994	0,12 %
8	less reliable gestational age evaluation method (LMP)	1834910	749144	29,0 %
9	induced (iatrogenic) delivery (or pregnancy before 1990)	1295490	539420	29,4 %
10	prelabor rupture of membranes (PROM)	1276244	19246	1,49 %
11	current Caesarean section	1273451	2793	0,22 %
12	previous Caesarean section	1206658	66793	5,25 %
13	self-reported maternal medical conditions	1188517	18141	1,50 %
14	pregnancy complications	1167804	20713	1,74 %
15	collapse rows to pregnancies (affects twins, triplets etc.)	1160063	7741	0,66 %
16	unclear twin/singleton assignment, triplets etc.	1159635	428	0,037 %
17	discordant maternal data in twin entries	1159542	93	0,008 %
18	birth weight is missing	1157760	1782	0,15 %
19	not the first pregnancy	535300	622460	53,8 %
20	maternal age <18 or >45	530888	4412	0,82 %

The authors would like to caution the reader in interpreting the "fraction removed" column: the applied filters overlap (i.e., are correlated), thus the fraction of removed pregnancies due to each filter is highly dependent on the order of applied filters and might not agree well with proportion of certain type of pregnancies estimated in other studies.
