# Supplementary material for: Uterine distention as a factor in birth timing: retrospective nationwide cohort study in Sweden
Source: BMJ Open. 2018 Oct 31;8(10):e022929. doi: 10.1136/bmjopen-2018-022929 (PMC6252709; doi:10.1136/bmjopen-2018-022929)
Supplement: Author's manuscript [file bmjopen-2018-022929.draft_revisions.pdf]

BMJ Open is committed to open peer review. As part of this commitment we make the peer review history of every article we publish publicly available.

When an article is published we post the peer reviewers' comments and the authors' responses online. We also post the versions of the paper that were used during peer review. These are the versions that the peer review comments apply to.

The versions of the paper that follow are the versions that were submitted during the peer review process. They are not the versions of record or the final published versions. They should not be cited or distributed as the published version of this manuscript.

BMJ Open is an open access journal and the full, final, typeset and author-corrected version of record of the manuscript is available on our site with no access controls, subscription charges or pay-per-view fees (http://bmjopen.bmj.com).

If you have any questions on BMJ Open's open peer review process please email info.bmjopen@bmj.com

BMJ Open

Evidence for gestation-imposed uterine distention determining the timing of birth

Journal:	*BMJ Open*
Manuscript ID	bmjopen-2018-022929
Article Type:	Research
Date Submitted by the Author:	14-Mar-2018
Complete List of Authors:	Bacelis, Jonas; Ostra sjukhuset, Obstetrics and Gynecology<br>Juodakis, Julius; Goteborgs universitet Institutionen for kliniska vetenskaper, Obstetrics and Gynecology<br>Adams Waldorf, Kristina; University of Washington, Obstetrics and Gynecology; Ostra sjukhuset, Obstetrics and Gynecology<br>Sengpiel, Verena; Ostra sjukhuset, Obstetrics and Gynecology<br>Muglia, Louis; Cincinnati Children's Hospital Medical Center, Center for Prevention of Preterm Birth<br>Zhang, Ge; Cincinnati Children's Hospital Medical Center, Center for Prevention of Preterm Birth; Cincinnati Children's Hospital Medical Center, Human Genetics Division<br>Jacobsson, Bo; Ostra sjukhuset, Obstetrics and Gynecology; Nasjonalt folkehelseinstitutt, Genetics and Bioinformatics
Keywords:	Gestational age at birth, Large for gestational age, Small for gestational age, Preterm delivery, Twins, Uterine distention

SCHOLARONE™
Manuscripts

only

TITLE PAGE

Title:
Evidence for gestation-imposed uterine distention determining the timing of birth

Corresponding author:
Jonas Bacelis
Diagnosvägen 15, 416 50, Gothenburg, Sweden
jonas.bacelis@gu.se, +46769259045

All co-authors:
Bacelis J[1$], Juodakis J[2], Adams Waldorf KM[1,3,4], Sengpiel V[1], Muglia LJ[5], Zhang G[5,6*], Jacobsson B[1,7*]

[1] Department of Obstetrics and Gynecology, Sahlgrenska University Hospital Östra, Gothenburg, Sweden
[2] Department of Obstetrics and Gynecology, Institute of Clinical Sciences, Sahlgrenska Academy, University of Gothenburg, Gothenburg, Sweden
[3] Department of Obstetrics and Gynecology, University of Washington, Seattle, Washington, USA
[4] Department of Global Health, University of Washington, Seattle, Washington, USA
[5] Center for Prevention of Preterm Birth, Perinatal Institute, Cincinnati Children's Hospital Medical Center and March of Dimes Prematurity Research Center Ohio Collaborative, Cincinnati, Ohio, USA
[6] Human Genetics Division, Cincinnati Children's Hospital Medical Center, Cincinnati, Ohio, USA
[7] Department of Genetics and Bioinformatics, Area of Health Data and Digitalisation, Norwegian Institute of Public Health, Oslo, Norway

[$] Corresponding author, [*] Co-senior authors

Word count:
2100 words

For peer review only - http://bmjopen.bmj.com/site/about/guidelines.xhtml

ABSTRACT

Objectives To determine whether uterine distention is associated with human pregnancy duration in a non-invasive observational setting.

Design Retrospective cohort study modeling uterine distention by interaction between maternal height and uterine load.

Setting The study is based on the 1990-2013 population data from all delivery units in Sweden.

Participants Uncomplicated first pregnancies of healthy Nordic-born mothers with spontaneous onset of labor. Pregnancies were classified as twin (n=2,846) or singleton (n=527,868). Singleton pregnancies were further classified as carrying a large for gestational age fetus (LGA, n=24,286) or small for gestational age fetus (SGA, n=33,780).

Outcome measures Statistical interaction between maternal height and uterine load categories (twin vs singleton pregnancies, and LGA vs SGA singleton pregnancies), where the outcome is pregnancy duration.

Results In all models, statistically significant interaction was found. Mothers carrying twins had 2.9 times larger positive linear effect of maternal height on gestational age than mothers carrying singletons (interaction p=5e-14). Similarly, the effect of maternal height was strongly modulated by the fetal growth rate in singleton pregnancies: the effect size of maternal height on gestational age in LGA pregnancies was 2.1 times larger than that in SGA pregnancies (interaction p<1e-11). Preterm birth odds ratio was 1.4 when the mother was short, and 2.8 when the fetus was extremely large for its gestational age; however, when both risk factors were present together, the odds ratio for preterm birth was larger than expected, 10.2 (interaction p<0.0005).

Conclusions Across all classes, maternal height was significantly associated with child's gestational age at birth. Interestingly, in short-statured women, spontaneous delivery occurred much earlier than expected when the uterine load was large (twins, LGA). The interaction between maternal height, uterine load size and gestational age at birth strongly suggests the effect of uterine distention imposed by fetal growth on birth timing.

Keywords Gestational age at birth, Large for gestational age, Small for gestational age, Preterm delivery, Twins, Uterine distention. (**Additional keywords:** Parturition, Pregnancy, Causality, Fetal growth rate, Gestational length, Interaction, Labor, Uterine load.)

For peer review only - http://bmjopen.bmj.com/site/about/guidelines.xhtml

STRENGTHS AND LIMITATIONS OF THE STUDY

- This is the first study to examine the effect of pregnancy-related uterine distention in a medical registry data (i.e., non-invasively).
- We used a large healthy homogeneous population and supported our findings with simulation analysis.
- To minimise confounding, uterine distention was modeled by two natural phenomena: twin pregnancies and singleton fetuses with very rapid growth.
- The study has implications to the design of future epidemiological, genetic, and proteomic preterm birth studies.
- A possible limitation of this study is the assumption that maternal height is a good proxy for uterine size.

INTRODUCTION

Preterm birth remains the leading cause of adverse outcomes of pregnancy. Globally preterm delivery is the leading cause of childhood mortality (under-five).[1] Indeed, the long-term health of the child is dependent to a great extent on the length of time spent in the mother's womb as a fetus. Worldwide, preterm delivery rates range from about 5% in some Northern European countries to 18% in Malawi.[2] Preterm labor is a complex phenotype thought to be triggered by several causes including microbial-induced inflammation, maternal stress, uterine distention, decidual haemorrhage and vascular diseases.[3] Complexities in the underlying pathophysiology of the preterm delivery process have been an obstacle to identify effective biomarkers and therapeutics.[4]

Epidemiological studies consistently reported that maternal height is positively associated with child's gestational age at birth, which has been replicated in different populations, ethnic

For peer review only - http://bmjopen.bmj.com/site/about/guidelines.xhtml

groups and time periods.[5-8] Using Mendelian randomisation methods, we demonstrated that this association is likely causal.[9] Building upon the causal association observed in uterine distention experiments in primates,[10] we hypothesed that birth is triggered by an interaction between uterine size and constantly increasing uterine load (fetal growth). In the current study, we test this hypothesis in humans by modeling the extreme of uterine distention in two natural "experiments": twin versus singleton pregnancy and in singletons with large and small fetuses for their gestational age.

For peer review only

For peer review only - http://bmjopen.bmj.com/site/about/guidelines.xhtml

METHODS

Study population

We investigated pregnancy records in the Swedish Medical Birth Register of mothers who were born in four Nordic countries: Sweden, Norway, Denmark and Finland. Since 1973, it is compulsory for every delivery unit in Sweden to provide the data to this register.

Only the first pregnancy of every mother was used, only with spontaneous onset of labor and only with live-born children. Due to the absence of indicators for spontaneous or iatrogenic onset of labor before the year 1990, only births from 1990 to 2013 were kept. Deliveries initiated by elective caesarean section or starting with prelabor rupture of membranes were removed. Further exclusions contained self-reported maternal medical conditions (diabetes, chronic hypertension, chronic kidney disease, inflammatory bowel disease, systemic lupus erythematosus), pregnancy complications reported in hospital records (placental disorders, placenta previa, early separation of placenta, antepartum haemorrhage, polyhydramnios, oligohydramnios) and estimated maternal body-mass index <15 or >45 kg/m^2, maternal age <18 or >45 years, higher order multiple births than twins.

Data quality and preparation

Maternal height

Pregnancies with maternal height values <140 cm and >200 cm were removed (0.01%), as well as height values that differed by more than 10 cm from other height values of the same mother in other pregnancies (0.12%).

Gestational age

Gestational age at birth (expressed in days) was estimated by midwives or obstetricians using the second-trimester ultrasonography method and supported by estimated gestational age at

For peer review only - http://bmjopen.bmj.com/site/about/guidelines.xhtml

birth reported by the delivery unit. We also excluded 0.01% of pregnancies that had birthweight values grossly inconsistent with gestational age.

Twin pregnancies

In order to ensure the initial assumption that twin pregnancies manifest larger uterine load than singleton pregnancies, we required that birthweight difference of two twins would be no larger than 2 SD of a mean intra-twin differences in a particular 7-day window of gestational age (**Sup Fig 1**). As a result of this filter, 174 (5.7%) twin pregnancies were removed.

Fetal Growth

The Medical Birth Register does not collect longitudinal data about the fetal growth (i.e., fetal growth curves). However, we used longitudinally derived intrauterine growth curves of healthy Scandinavian pregnancies with term deliveries derived my Marsal et al.[11] in order to convert birthweight, fetal sex and gestational age at birth data into birthweight Z-scores that can be interpreted as being proportional to the fetal growth rate. The newborns with a Z-score larger than 1.5 in the singleton cohort were classified as large for gestational age (LGA), and newborns with a Z-score lower than -1.5 in the singleton cohort were classified as small for gestational age (SGA), **Sup Fig 2**.

Data analysis

We explored how uterine load modulates the effect of maternal height on child's gestational age at birth. We used two models of uterine load: Model 1 with twin versus singleton pregnancies, and Model 2 with LGA versus SGA pregnancies (all singleton). We assumed that at the time of delivery, twins and LGA singletons manifest a larger volumetric uterine load than singletons and SGA singletons, respectively.

In both models, we ran multiple linear regression with child's gestational age at birth (GA)

For peer review only

For peer review only - http://bmjopen.bmj.com/site/about/guidelines.xhtml

being dependent on continuous maternal height (MH), uterine load (UL) categories, and their interaction, where large uterine load was coded as "1" (twins or LGA) and small uterine load coded as "0" (singletons or SGA):

$$GA = \beta_0 + \beta_1 * MH + \beta_2 * UL + \beta_3 * MH * UL + \varepsilon$$

Additionally, we used logictic regression to investigate a the same phenomenon in the clinical phenotype - preterm delivery (PTD; birth occurring earlier than 259 days of gestation; standard definition). In this analysis we used extreme dichotomous definitions of maternal stature and uterine load. Short stature was defined by maternal height <161 cm (10th percentile, rounded to integers). In Model 1, high volumetric uterine load was assigned to twin pregnancies, and in Model 2, high volumetric uterine load was assigned to singleton pregnancies with extreme fetal growth, defined by fetus having a birthweight Z-score larger than 2.75 SD. Statistical significance was reported as interaction term P-value.

All statistical analyses were performed with R software for statistical computing (version 3.3.1). The analytical code can be found on github.com/PerinatalLab/MatHeight-GestAge.

Simulation

We modeled the hypothesized phenomenon in a computer simulation to make sure that the used assumptions were sufficient to produce results similar to the real data-generated results.

Each pregnancy was assigned a randomly generated maternal uterine size and fetal growth rate. Maternal height was derived as a correlate of uterine size. As simulation time progressed (i.e., "gestation"), the births were triggered by either of two competing risks, whichever occurred first: baseline or the fetal size reaching the uterine size capacity. Gestational age at birth was recorded for each pregnancy. Generated data was used to test the interaction effect in the same way as described in the Data Analysis.

For peer review only - http://bmjopen.bmj.com/site/about/guidelines.xhtml

Patient involvement

Patients were not involved in setting the research questions or planning the study.

Investigators do not know the identity of study participants.

For peer review only - http://bmjopen.bmj.com/site/about/guidelines.xhtml

RESULTS

Our study, investigating uncomplicated first pregnancies of healthy Nordic-born mothers with spontaneous onset of labor, contained 2 846 twin and 527 868 singleton pregnancies. Singleton pregnancies carried 24 286 LGA fetuses and 33 780 SGA fetuses (**Fig 1**).

Gestational age

In Model 1, we investigated the relationship between child's gestational age and maternal height in two categories: women with twin and singleton pregnancies. A clear interaction pattern was detected (**Fig 2, Panel 1**), with timing of birth being more sensitive to maternal height in twin pregnancies than in singleton pregnancies. Shorter mothers with twin pregnancies deliver earlier than it would be expected based on the trend in singleton pregnancies and the mean difference between gestational age in singleton and twin pregnancies. The coefficients estimated in multiple linear regression (**Table 1**) show that maternal height has 2.9 times larger positive linear effect on gestational age in twin pregnancies ($\beta_3+\beta_1$) than it has in singleton pregnancies (β_1). The observed effect-modulating behavior of uterine load (β_3 coefficients) is statistically significant, interaction P=5e-14.

Table 1. Estimated effect sizes in Model 1 from multiple linear regression. Estimate is expressed in days of gestation. MH - maternal height (cm), UL - uterine load (singletons=0, twins=1). SE - standard error, P - statistical significance.

Coefficient	Estimate	SE	P
β_0 (intercept)	258.2	0.412	<1E-150
β_1 (MH)	0.131	0.002	<1E-150
β_2 (UL)	-69.20	5.619	8E-35
β_3 (MH x UL)	0.252	0.033	5E-14

In Model 2, we observed similar results: a clear interaction pattern was observed (**Fig 2, Panel 2**), where timing of birth was more sensitive to maternal height in singleton pregnancies with LGA fetuses than singleton pregnancies with SGA fetuses. Shorter mothers

For peer review only - http://bmjopen.bmj.com/site/about/guidelines.xhtml

with LGA pregnancies deliver earlier than it would be expected based on the trend in SGA pregnancies and the mean difference between gestational age in SGA and LGA pregnancies. The coefficients estimated in multiple linear regression (**Table 2**) show that maternal height has 2.1 times larger positive linear effect on gestational age in LGA pregnancies (β_3+β_1) than it has in SGA pregnancies (β_1). The observed effect-modulating behaviour of volumetric uterine load (β_3 coefficient) is statistically significant, interaction P=1e-11.

Table 2. Estimated effect sizes in Model 2 from multiple linear regression. Estimate is expressed in days of gestation. MH - maternal height (cm), UL - uterine load (SGA=0, LGA=1). SE - standard error, P - statistical significance.

Coefficient	Estimate	SE	P
β_0 (intercept)	264.6	1.646	<1E-150
β_1 (MH)	0.096	0.0099	5E-22
β_2 (UL)	-20.13	2.598	1E-14
β_3 (MH x UL)	0.105	0.0154	1E-11

Preterm birth

In additional analyses focusing on the clinical phenotype preterm birth, we observed that preterm birth was more prevalent in the risk group of "short mother and high uterine load" than it would be expected without synergistic interaction between the two risk factors "short mother" and "high uterine load".

In Model 1, the odds ratio for preterm birth in the "short mother carrying twins" risk group was larger than it would be expected without interaction between the risk factors "short mother" and "twin pregnancy" (**Fig 3, Panel 1**). However, the interaction term in the logistic regression was not statistically significant (P=0.1).

In Model 2, the odds ratio for preterm birth in the "short mother with extreme fetal growth" risk group was larger than it would be expected without interaction (**Fig 3, Panel 2**). The corresponding interaction term in the logistic regression was statistically significant

For peer review only - http://bmjopen.bmj.com/site/about/guidelines.xhtml

(P=0.0005).

Simulation

In a simulation experiment, we tested whether the observed interaction pattern (**Fig 2, Panel 2**) could be qualitatively reproduced, if birth events were indeed to be triggered by fetal size reaching a certain threshold determined by height-correlated uterine size. The observed interaction pattern was replicated in a simulation (**Sup Fig 3**), where birth events were initiated by two competing risks: baseline (unknown environmental and genetic factors) and interaction between fetal growth rate and height-correlated uterine size. Similarly to **Fig 2 (Panel 2)**, LGA pregnancies had a steeper slope than SGA pregnancies.

For peer review only

DISCUSSION

In this retrospective cohort study we investigated first pregnancies of healthy Nordic-born mothers delivering in Sweden during the period 1990-2013 with spontaneous onset of labor. We found that child's gestational age is more sensitive to maternal height in pregnancies with larger uterine load than in pregnancies with smaller uterine load: the effect of maternal height is significantly larger in twin versus singleton pregnancies, and in singleton pregnancies with fast- vs slow-growing fetus. The observed statistical interaction is likely a manifestation of biomechanical interplay between uterine size and uterine load (fetal size) in time. The labor in uncomplicated spontaneous delivery is likely triggered by uterine distention, which intensifies with the progression of pregnancy and reaches its peak when fetal size approaches the uterine size.

Our results support the previous causality claims for maternal height[9] and iatrogenic uterine stretch determining the timing of birth[10,12,13], and suggests that gestation-imposed uterine stretch also affects birth timing in spontaneous human pregnancies. An alternative explanation could be related to metabolic constraint imposed by maternal energy expenditure and fetal energy demand over gestational time,[14] as twins and LGA singletons require more energy to support their growth and maternal basal metabolism is related to height.[15]

Sensitivity analyses showed that the interaction effect is most clearly observed in the first-pregnancy (parity = 0) but not when parity was higher. Future studies could explore the changes/adaptations in myometrium after the first delivery.

The biomechanical explanation of timing of birth will benefit genomic and proteomic association studies of preterm birth by increasing statistical power: our results implicate candidate genes and candidate proteins from inflammation-mediated biochemical pathways which are excited by uterine distention.[10]

For peer review only - http://bmjopen.bmj.com/site/about/guidelines.xhtml

The future predictive algorithms could take into account fetal growth speed and uterine size while trying to classify pregnancies as being low or high risk for preterm birth.

CONCLUSION

The observed interactions support the hypothesis of uterine distention playing a causal role in determining the timing of birth, at the same time giving support to the causal effect of maternal height, uterine load, uterine size.

ACKNOWLEDGMENTS, COMPETING INTERESTS, FUNDING AND OTHER

Acknowledgements We are grateful to The Swedish National Board of Health and Welfare (*Socialstyrelsen*) who manages the Swedish Medical Birth Register.

Contributors JB and GZ - study design; BJ and VS - data acquisition; JB and JJ - data cleaning and figures; JB and GZ - data analysis and interpretation, KAW - literature search; JB and KAW- writing; BJ, JJ, LJM, VS, GZ - critical revision. All authors approved the final version to be submitted for publication.

Funding This work was supported by Swedish government grants to researchers in the public health sector (ALFGBG-717501, ALFGBG-507701, ALFGBG-426411), The Swedish Research Council (2015-02559) and March of Dimes Foundation (21-FY16-121).

Competing interests None declared.

Ethics approval The work was approved by the Regional Ethical Review Board in the Western Health Care Region of Sweden (Dnr:968-14).

Provenance and peer review Not commissioned; externally peer reviewed.

Data sharing statement Researchers can apply for data access by contacting The Swedish National Board of Health and Welfare.

For peer review only - http://bmjopen.bmj.com/site/about/guidelines.xhtml

REFERENCES

1. Liu L, Oza S, Hogan D, et al. Global, regional, and national causes of under-5 mortality in 2000-15: an updated systematic analysis with implications for the Sustainable Development Goals. *Lancet* 2016;388(10063):3027-35. doi: 10.1016/S0140-6736(16)31593-8 [published Online First: 2016/11/15].

2. Blencowe H, Cousens S, Oestergaard MZ, et al. National, regional, and worldwide estimates of preterm birth rates in the year 2010 with time trends since 1990 for selected countries: a systematic analysis and implications. *Lancet* 2012;379(9832):2162-72. doi: 10.1016/S0140-6736(12)60820-4 [published Online First: 2012/06/12].

3. Romero R, Dey SK, Fisher SJ. Preterm labor: one syndrome, many causes. *Science* 2014;345(6198):760-5. doi: 10.1126/science.1251816 [published Online First: 2014/08/16].

4. Iams JD, Romero R, Culhane JF, et al. Primary, secondary, and tertiary interventions to reduce the morbidity and mortality of preterm birth. *Lancet* 2008;371(9607):164-75. doi: 10.1016/S0140-6736(08)60108-7 [published Online First: 2008/01/15].

5. Chan BC, Lao TT. Maternal height and length of gestation: does this impact on preterm labour in Asian women? *Aust N Z J Obstet Gynaecol* 2009;49(4):388-92. doi: 10.1111/j.1479-828X.2009.01006.x [published Online First: 2009/08/22].

6. Dickey RP, Xiong X, Xie Y, et al. Effect of maternal height and weight on risk for preterm singleton and twin births resulting from IVF in the United States, 2008-2010. *Am J Obstet Gynecol* 2013;209(4):349 e1-6. doi: 10.1016/j.ajog.2013.05.052 [published Online First: 2013/06/04].

7. Derraik JG, Lundgren M, Cutfield WS, et al. Maternal Height and Preterm Birth: A Study on 192,432 Swedish Women. *PLoS One* 2016;11(4):e0154304. doi: 10.1371/journal.pone.0154304 [published Online First: 2016/04/23].

8. Morken NH, Kallen K, Jacobsson B. Predicting risk of spontaneous preterm delivery in women with a singleton pregnancy. *Paediatric and perinatal epidemiology* 2014;28(1):11-22. doi: 10.1111/ppe.12087 [published Online First: 2013/10/15].

9. Zhang G, Bacelis J, Lengyel C, et al. Assessing the Causal Relationship of Maternal Height on Birth Size and Gestational Age at Birth: A Mendelian Randomization Analysis. *PLoS medicine* 2015;12(8):e1001865. doi: 10.1371/journal.pmed.1001865 [published Online First: 2015/08/19].

10. Adams Waldorf KM, Singh N, Mohan AR, et al. Uterine overdistention induces preterm labor mediated by inflammation: observations in pregnant women and nonhuman primates. *Am J Obstet Gynecol* 2015;213(6):830 e1-30 e19. doi: 10.1016/j.ajog.2015.08.028 [published Online First: 2015/08/19].

11. Marsal K, Persson PH, Larsen T, et al. Intrauterine growth curves based on ultrasonically estimated foetal weights. *Acta Paediatr* 1996;85(7):843-8. [published Online First: 1996/07/01].

12. Manabe Y, Mori T, Yoshida Y. Decidual morphology and F prostaglandin in amniotic fluid in stretch-induced abortion. *Obstet Gynecol* 1984;64(5):661-5. [published Online First: 1984/11/01].

For peer review only - http://bmjopen.bmj.com/site/about/guidelines.xhtml

13. Manabe Y, Manabe A, Takahashi A. Effect of indomethacin on stretch-induced uterine activity in the post-partum. *Prostaglandins* 1983;25(5):653-9. [published Online First: 1983/05/01].

14. Dunsworth HM, Warrener AG, Deacon T, et al. Metabolic hypothesis for human altriciality. *Proc Natl Acad Sci U S A* 2012;109(38):15212-6. doi: 10.1073/pnas.1205282109 [published Online First: 2012/08/31].

15. Harris JA, Benedict FG. A Biometric Study of Human Basal Metabolism. *Proc Natl Acad Sci U S A* 1918;4(12):370-3. [published Online First: 1918/12/01].

FIGURE LEGENDS

Fig 1. Flowchart for selection of analysed pregnancies. AGA - child's birthweight is appropriate for gestational age; LGA and SGA - birthweight is large and small for gestational age, respectively. BMI - Body-mass index. LMP - last menstrual period. Two grey boxes represent the analysis in Model 1, two yellow boxes represent analysis in Model 2.

Fig 2. Panel 1) Interaction pattern in Model 1, where uterine load is modelled as twin vs singleton pregnancies. **Panel 2)** Interaction pattern in Model 2, where uterine load is modelled as too large vs too small birthweight for gestational age in singleton pregnancies (LGA and SGA, respectively). Observations are grouped based on maternal height bins. CI - confidence interval. N - count of observations used to estimate mean.

Fig 3. Preterm birth in short mothers with: **Panel 1)** twin pregnancies, **Panel 2)** extreme fetal growth. Short stature is defined as <161cm. Preterm birth is defined as delivery before 259 days of gestation. Extreme fetal growth is defined as >2.75 birthweight Z-score (adjusted for gestational age). The grey angular lines denote 95% confidence interval for expected odds ratio in stratum D, estimated from odds ratios in strata B and C with assumption of no interaction. In Panel 1, odds ratio in stratum D is larger than expected due to interaction between maternal height and fetal growth (interaction P=0.1, logistic regression). In Panel 2, odds ratio in stratum D is significantly larger than expected due to interaction between maternal height and fetal growth (interaction P<0.0005, logistic regression).

For peer review only - http://bmjopen.bmj.com/site/about/guidelines.xhtml

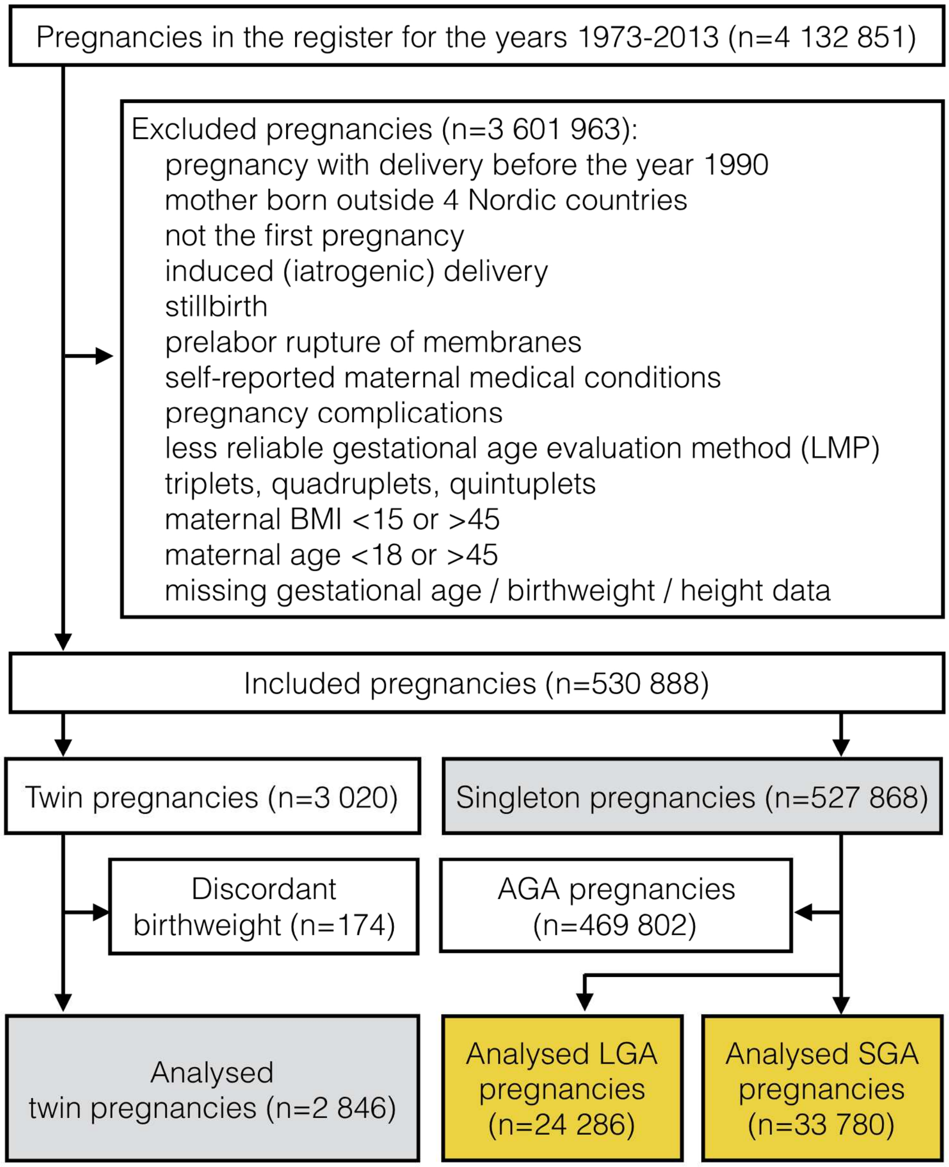

Figure 1. Flowchart for selection of analysed pregnancies. AGA - child's birthweight is appropriate for gestational age; LGA and SGA - birthweight is large and small for gestational age, respectively. BMI - Body-mass index. LMP - last menstrual period. Two grey boxes represent the analysis in Model 1, two yellow boxes represent analysis in Model 2.

185x226mm (300 x 300 DPI)

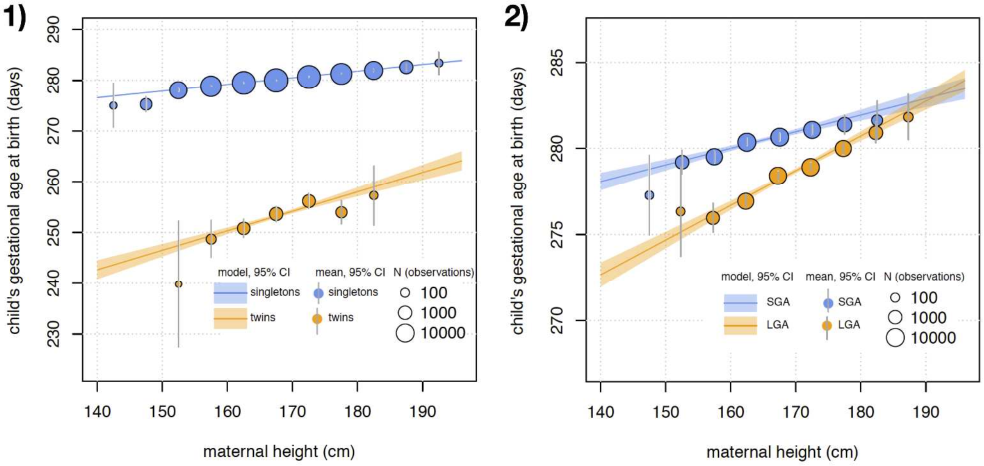

Figure 2. **Panel 1)** Interaction pattern in Model 1, where uterine load is modelled as twin vs singleton pregnancies. **Panel 2)** Interaction pattern in Model 2, where uterine load is modelled as too large vs too small birthweight for gestational age in singleton pregnancies (LGA and SGA, respectively). Observations are grouped based on maternal height bins. CI - confidence interval. N - count of observations used to estimate mean.

357x173mm (300 x 300 DPI)

review only

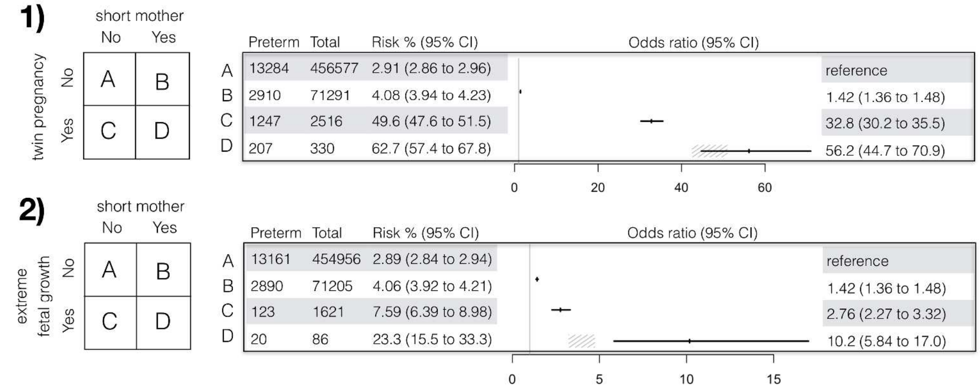

Figure 3. Preterm birth in short mothers with: **Panel 1)** twin pregnancies, **Panel 2)** extreme fetal growth. Short stature is defined as <161cm. Preterm birth is defined as delivery before 259 days of gestation. Extreme fetal growth is defined as >2.75 birthweight Z-score (adjusted for gestational age). The grey angular lines denote 95% confidence interval for expected odds ratio in stratum D, estimated from odds ratios in strata B and C with assumption of no interaction. In Panel 1, odds ratio in stratum D is larger than expected due to interaction between maternal height and fetal growth (interaction P=0.1, logistic regression). In Panel 2, odds ratio in stratum D is significantly larger than expected due to interaction between maternal height and fetal growth (interaction P<0.0005, logistic regression).

338x140mm (300 x 300 DPI)

Supplementary Figures

"Evidence for gestation-imposed uterine distention determining the timing of birth"

2018-03-13, Jonas Bacelis et al.

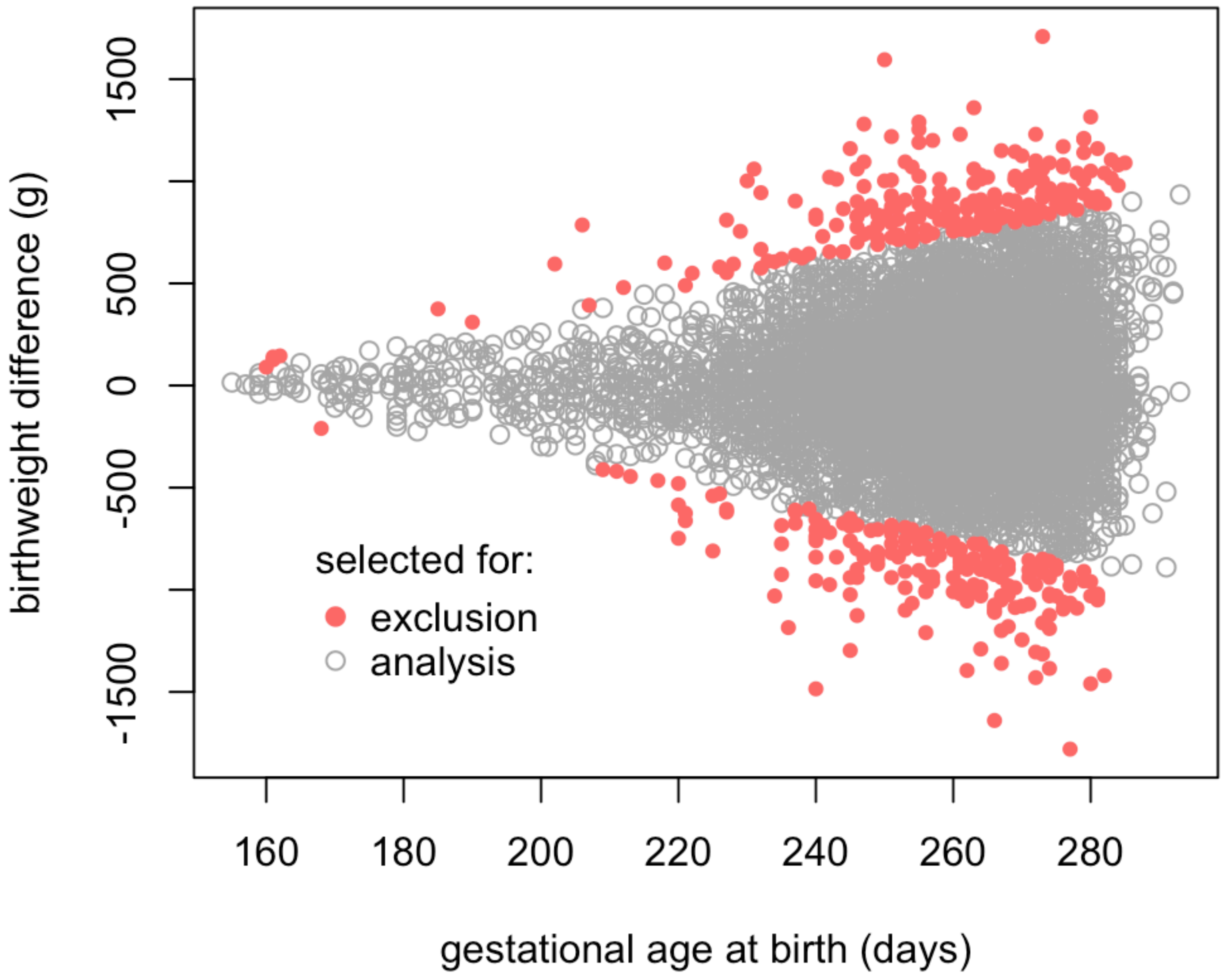

Sup Fig 1 l Selection of twin pregnancies. Pregnancies were excluded if birthweight of two twins differed by more than 2 SD of all intra-twin differences in that gestational age window. Pregnancies with thus discordant twin birthweights (n=174) were excluded from analyses.

Supplementary Figures

"Evidence for gestation-imposed uterine distention determining the timing of birth"
2018-03-13, Jonas Bacelis et al.

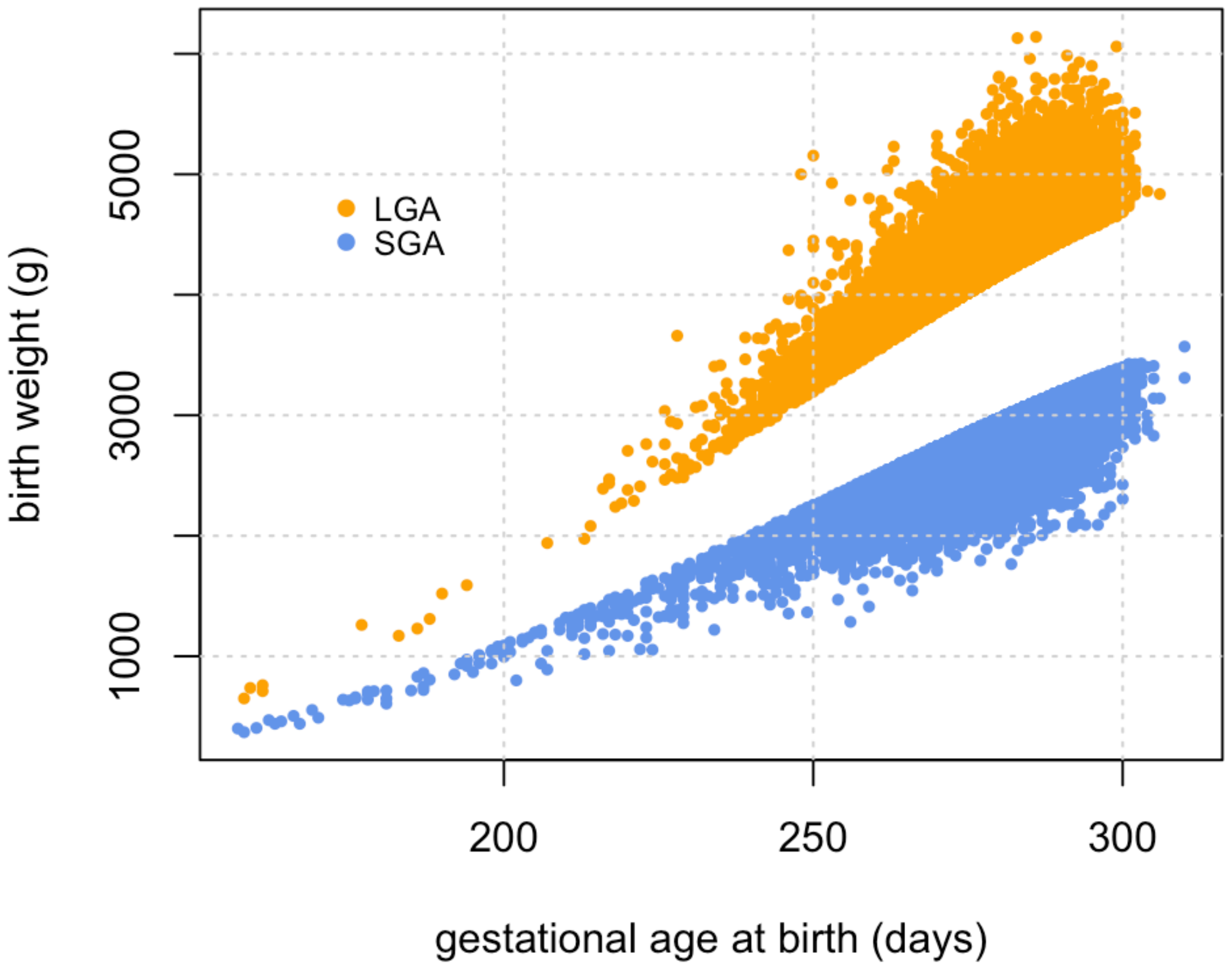

Sup Fig 2 I Classifying singleton births as small (SGA) or large (LGA) for gestational age using birthweight Z-score <-1.5 and >1.5, respectively. Birthweight Z-score was estimated from longitudinally derived intra-uterine growth curves in heathy term pregnancies using polynomial regression equation from Marsal et al., which takes into account child's sex, birthweight, and ultrasound-determined gestational age at birth.

Supplementary Figures

"Evidence for gestation-imposed uterine distention determining the timing of birth"
2018-03-13, Jonas Bacelis et al.

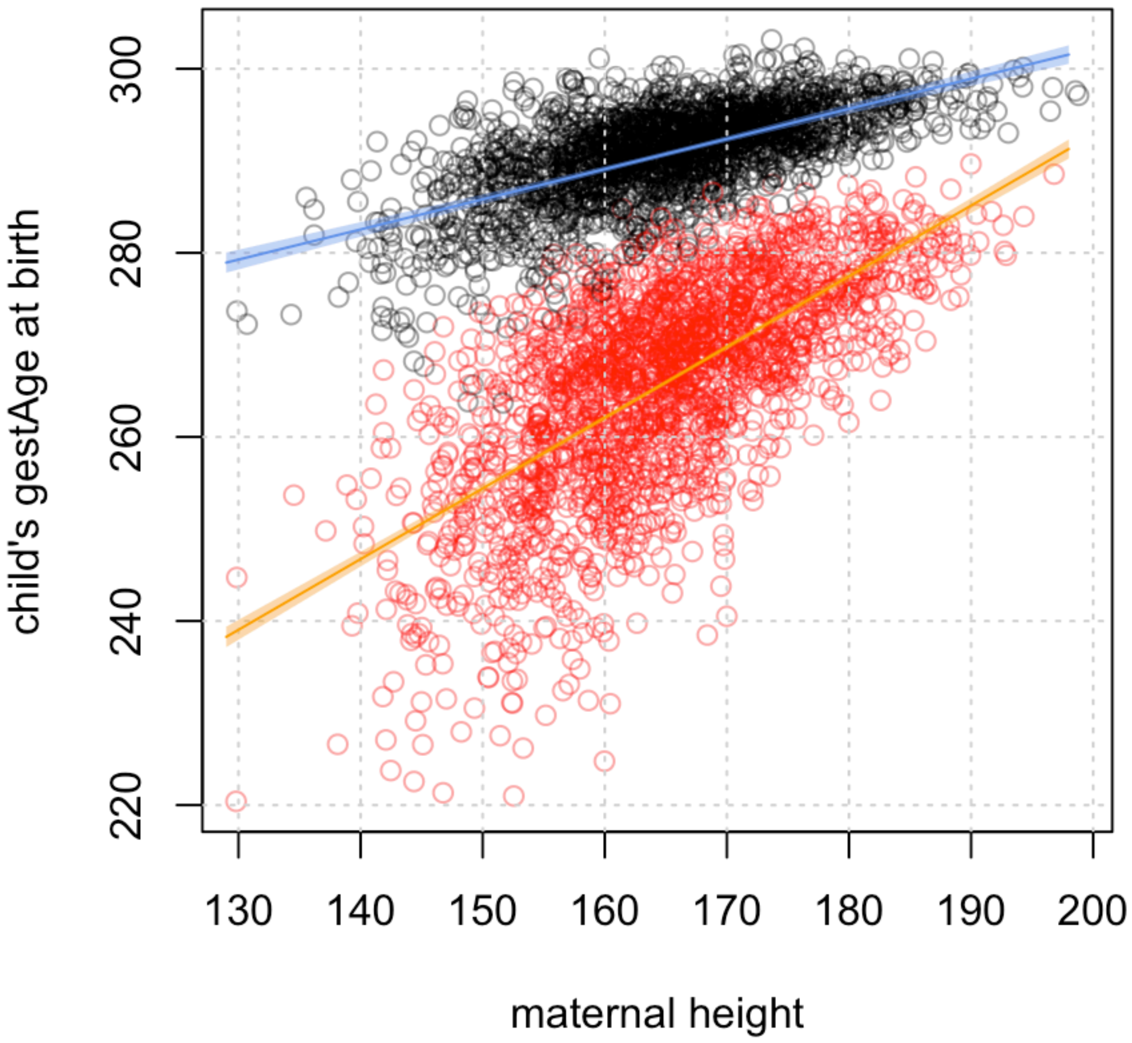

Sup Fig 3 l Simulation of interaction effect. The birth events (gestational age at birth) are triggered by fetal size reaching the uterine size capacity. Fetal size is determined by randomly assigned fetal growth rate, maternal height is correlated to randomly assigned maternal uterine size. Pregnancies with slow fetal growth are depicted in black circles and blue regression line; pregnancies with fast fetal growth are depicted in red circles and orange regression line.

STROBE Statement—checklist of items that should be included in reports of observational studies

	Item No.	Recommendation	Page No.	Relevant text from manuscript
**Title and abstract**	1	(*a*) Indicate the study's design with a commonly used term in the title or the abstract	2	"Retrospective cohort study.."
		(*b*) Provide in the abstract an informative and balanced summary of what was done and what was found	2	"Statistical interaction between maternal height and uterine load categories.."
**Introduction**				
Background/rationale	2	Explain the scientific background and rationale for the investigation being reported	3	"..preterm delivery is the leading cause of childhood mortality.."
Objectives	3	State specific objectives, including any prespecified hypotheses	4	"..we hypothesed that birth is triggered by an interaction between uterine size and constantly increasing uterine load.."
**Methods**				
Study design	4	Present key elements of study design early in the paper	5	[pregnancies in Sweden of] "..mothers who were born in four Nordic countries: Sweden, Norway, Denmark and Finland."
Setting	5	Describe the setting, locations, and relevant dates, including periods of recruitment, exposure, follow-up, and data collection	5	"..Swedish Medical Birth Register.." "..1990 to 2013.." "..the first pregnancy of every mother was used.."
Participants	6	(*a*) *Cohort study*—Give the eligibility criteria, and the sources and methods of selection of participants. Describe methods of follow-up *Case-control study*—Give the eligibility criteria, and the sources and methods of case	5	"Deliveries initiated by elective caesarean section or starting with prelabor rupture of

For peer review only - http://bmjopen.bmj.com/site/about/guidelines.xhtml

For peer review only

		ascertainment and control selection. Give the rationale for the choice of cases and controls<br>*Cross-sectional study*—Give the eligibility criteria, and the sources and methods of selection of participants		membranes were removed. Further exclusions contained self-reported maternal medical conditions [...], pregnancy complications.."
		(*b*) *Cohort study*—For matched studies, give matching criteria and number of exposed and unexposed<br>*Case-control study*—For matched studies, give matching criteria and the number of controls per case		
Variables	7	Clearly define all outcomes, exposures, predictors, potential confounders, and effect modifiers. Give diagnostic criteria, if applicable	6<br>6	"..child's gestational age at birth (GA) being dependent on continuous maternal height (MH), uterine load (UL) categories.."<br>"..uterine load: [1] twin versus singleton pregnancies, and [2] LGA versus SGA pregnancies.."
Data sources/ measurement	8*	For each variable of interest, give sources of data and details of methods of assessment (measurement). Describe comparability of assessment methods if there is more than one group	*5,6*	"Maternal height.."<br>"Gestational age.."<br>"Twin pregnancies.."<br>"Fetal Growth.."
Bias	9	Describe any efforts to address potential sources of bias	6<br>7	"We used two [very different] models of uterine load.."<br>"..computer simulation [was used] to make sure that the used assumptions were sufficient.."
Study size	10	Explain how the study size was arrived at	5	Section "Study Population" and below

Continued on next page

For peer review only - http://bmjopen.bmj.com/site/about/guidelines.xhtml

Quantitative variables	11	Explain how quantitative variables were handled in the analyses. If applicable, describe which groupings were chosen and why	7	"..continuous maternal height (MH), uterine load (UL) categories.." and formula below
Statistical methods	12	(*a*) Describe all statistical methods, including those used to control for confounding	6<br>7	"multiple linear regression.."<br>"..logictic regression.."
		(*b*) Describe any methods used to examine subgroups and interactions	7	the whole page
		(*c*) Explain how missing data were addressed	Fig1	excluded pregnancies with "missing gestational age / birthweight / height data"
		(*d*) *Cohort study*—If applicable, explain how loss to follow-up was addressed<br>*Case-control study*—If applicable, explain how matching of cases and controls was addressed<br>*Cross-sectional study*—If applicable, describe analytical methods taking account of sampling strategy		not applicable
		(*e*) Describe any sensitivity analyses	12	"Sensitivity analyses showed.."
**Results**				
Participants	13*	(a) Report numbers of individuals at each stage of study—eg numbers potentially eligible, examined for eligibility, confirmed eligible, included in the study, completing follow-up, and analysed	Fig1	
		(b) Give reasons for non-participation at each stage	Fig1	
		(c) Consider use of a flow diagram	Fig1	
Descriptive data	14*	(a) Give characteristics of study participants (eg demographic, clinical, social) and information on exposures and potential confounders	5	"mothers who were born in four Nordic countries: Sweden, Norway, Denmark and Finland"<br>"Further exclusions contained.."
		(b) Indicate number of participants with missing data for each variable of interest	Fig1	[no individuals with missing data]
		(c) *Cohort study*—Summarise follow-up time (eg, average and total amount)		not aplicable
Outcome data	15*	*Cohort study*—Report numbers of outcome events or summary measures over time		not aplicable (our outcome is interaction three variables)
		Case-control study—Report numbers in each exposure category, or summary measures of exposure	Fig3	somewhat applicable
		Cross-sectional study—Report numbers of outcome events or summary measures		
Main results	16	(*a*) Give unadjusted estimates and, if applicable, confounder-adjusted estimates and their precision (eg, 95% confidence interval). Make clear which confounders were adjusted for and why they were		Not applicable. Confounder adjustment would be too

For peer review only - http://bmjopen.bmj.com/site/about/guidelines.xhtml

	included		complicated to interpret in this particular study investigating interaction effect. We used different approach (two different models of phenomenon and simulation) to address this problem.
	(*b*) Report category boundaries when continuous variables were categorized	6	"The newborns with a Z-score larger than 1.5 in the singleton cohort were classified as large for gestational age (LGA), and newborns with a Z-score lower than -1.5 in the singleton cohort were classified as small for gestational age (SGA).."
	(*c*) If relevant, consider translating estimates of relative risk into absolute risk for a meaningful time period		not relevant

Continued on next page

For peer review only - http://bmjopen.bmj.com/site/about/guidelines.xhtml

Other analyses	17	Report other analyses done—eg analyses of subgroups and interactions, and sensitivity analyses	12	"Sensitivity analyses showed.."
**Discussion**				
Key results	18	Summarise key results with reference to study objectives	12	"..the observed statistical interaction is likely a manifestation of biomechanical interplay between uterine size and uterine load (fetal size) in time.."
Limitations	19	Discuss limitations of the study, taking into account sources of potential bias or imprecision. Discuss both direction and magnitude of any potential bias	12<br>12<br>3	"..An alternative explanation.."<br>"..not when parity was higher.."<br>"..possible limitation of this study.."
Interpretation	20	Give a cautious overall interpretation of results considering objectives, limitations, multiplicity of analyses, results from similar studies, and other relevant evidence	13	"The observed interactions support the hypothesis .."
Generalisability	21	Discuss the generalisability (external validity) of the study results	12	"..not when parity was higher.."
**Other information**				
Funding	22	Give the source of funding and the role of the funders for the present study and, if applicable, for the original study on which the present article is based	13	"Funding.."

*Give information separately for cases and controls in case-control studies and, if applicable, for exposed and unexposed groups in cohort and cross-sectional studies.

Note: An Explanation and Elaboration article discusses each checklist item and gives methodological background and published examples of transparent reporting. The STROBE checklist is best used in conjunction with this article (freely available on the Web sites of PLoS Medicine at http://www.plosmedicine.org/, Annals of Internal Medicine at http://www.annals.org/, and Epidemiology at http://www.epidem.com/). Information on the STROBE Initiative is available at www.strobe-statement.org.

For peer review only - http://bmjopen.bmj.com/site/about/guidelines.xhtml

BMJ Open

Uterine distention as a factor in birth timing: retrospective nationwide cohort study in Sweden

Journal:	*BMJ Open*
Manuscript ID	bmjopen-2018-022929.R1
Article Type:	Research
Date Submitted by the Author:	05-Sep-2018
Complete List of Authors:	Bacelis, Jonas; Sahlgrenska University Hospital Östra, Obstetrics and Gynecology<br>Juodakis, Julius; University of Gothenburg, Sahlgrenska Academy, Institute of Clinical Sciences, dept. Obstetrics and Gynecology<br>Adams Waldorf, Kristina; University of Washington, Obstetrics and Gynecology; Sahlgrenska University Hospital Östra, Obstetrics and Gynecology<br>Sengpiel, Verena; Sahlgrenska University Hospital Östra, Obstetrics and Gynecology<br>Muglia, Louis; Cincinnati Children's Hospital Medical Center, Human Genetics Division; Cincinnati Children's Hospital Medical Center, Center for Prevention of Preterm Birth<br>Zhang, Ge; Cincinnati Children's Hospital Medical Center, Human Genetics Division; Cincinnati Children's Hospital Medical Center, Center for Prevention of Preterm Birth<br>Jacobsson, Bo; Sahlgrenska University Hospital Östra, Obstetrics and Gynecology; Nasjonalt folkehelseinstitutt, Genetics and Bioinformatics
**Primary Subject Heading**:	Obstetrics and gynaecology
Secondary Subject Heading:	Epidemiology
Keywords:	Gestational age at birth, Large for gestational age, Small for gestational age, Preterm delivery, Twins, Uterine distention

SCHOLARONE™
Manuscripts

TITLE PAGE

Title:

Uterine distention as a factor in birth timing: retrospective nationwide cohort study in Sweden

Corresponding author:

Jonas Bacelis

Diagnosvägen 15, 416 50, Gothenburg, Sweden

jonas.bacelis@gu.se, +46769259045

All co-authors:

Bacelis J[1$], Juodakis J[2], Adams Waldorf KM[1,3,4], Sengpiel V[1], Muglia LJ[5,6], Zhang G[5,6*], Jacobsson B[1,7*]

[1] Department of Obstetrics and Gynecology, Sahlgrenska University Hospital Östra, Gothenburg, Sweden

[2] Department of Obstetrics and Gynecology, Institute of Clinical Sciences, Sahlgrenska Academy, University of Gothenburg, Gothenburg, Sweden

[3] Department of Obstetrics and Gynecology, University of Washington, Seattle, Washington, USA

[4] Department of Global Health, University of Washington, Seattle, Washington, USA

[5] Human Genetics Division, Cincinnati Children's Hospital Medical Center, Cincinnati, Ohio, USA

[6] Center for Prevention of Preterm Birth, Perinatal Institute, Cincinnati Children’s Hospital Medical Center and March of Dimes Prematurity Research Center Ohio Collaborative, Cincinnati, Ohio, USA

[7] Department of Genetics and Bioinformatics, Area of Health Data and Digitalisation, Norwegian Institute of Public Health, Oslo, Norway

[$] Corresponding author, [*] Co-senior authors

Word count:

2200 words

For peer review only

For peer review only - http://bmjopen.bmj.com/site/about/guidelines.xhtml

ABSTRACT

Objectives To determine whether uterine distention is associated with human pregnancy duration in a non-invasive observational setting.

Design Retrospective cohort study modeling uterine distention by interaction between maternal height and uterine load.

Setting The study is based on the 1990-2013 population data from all delivery units in Sweden.

Participants Uncomplicated first pregnancies of healthy Nordic-born mothers with spontaneous onset of labor. Pregnancies were classified as twin (n=2,846) or singleton (n=527,868). Singleton pregnancies were further classified as carrying a large for gestational age fetus (LGA, n=24,286) or small for gestational age fetus (SGA, n=33,780).

Outcome measures Statistical interaction between maternal height and uterine load categories (twin vs singleton pregnancies, and LGA vs SGA singleton pregnancies), where the outcome is pregnancy duration.

Results In all models, statistically significant interaction was found. Mothers carrying twins had 2.9 times larger positive linear effect of maternal height on gestational age than mothers carrying singletons (interaction p=5e-14). Similarly, the effect of maternal height was strongly modulated by the fetal growth rate in singleton pregnancies: the effect size of maternal height on gestational age in LGA pregnancies was 2.1 times larger than that in SGA pregnancies (interaction p<1e-11). Preterm birth odds ratio was 1.4 when the mother was short, and 2.8 when the fetus was extremely large for its gestational age; however, when both risk factors were present together, the odds ratio for preterm birth was larger than expected, 10.2 (interaction p<0.0005).

Conclusions Across all classes, maternal height was significantly associated with child's gestational age at birth. Interestingly, in short-statured women, spontaneous delivery occurred much earlier than expected when the uterine load was large (twins, LGA). The interaction between maternal height, uterine load size and gestational age at birth strongly suggests the effect of uterine distention imposed by fetal growth on birth timing.

Keywords Gestational age at birth, Large for gestational age, Small for gestational age, Preterm delivery, Twins, Uterine distention. (**Additional keywords:** Parturition, Pregnancy, Causality, Fetal growth rate, Gestational length, Interaction, Labor, Uterine load.)

STRENGTHS AND LIMITATIONS OF THE STUDY

- This is the first study to examine the effect of pregnancy-related uterine distention in a medical registry data (i.e., non-invasively).
- We used a large healthy homogeneous population and supported our findings with simulation analysis.
- To minimize confounding, uterine distention was modeled by two natural phenomena: twin pregnancies and singleton fetuses with very rapid growth.
- The study has implications to the design of future epidemiological, genetic, and proteomic preterm birth studies.
- A possible limitation of this study is the assumption that maternal height is a good proxy for uterine size.

For peer review only

For peer review only - http://bmjopen.bmj.com/site/about/guidelines.xhtml

INTRODUCTION

Preterm birth remains the leading cause of adverse outcomes of pregnancy. Globally preterm delivery is the leading cause of childhood mortality (under-five).[1] Indeed, the long-term health of the child is dependent to a great extent on the length of time spent in the mother's womb as a fetus. Worldwide, preterm delivery rates range from about 5% in some Northern European countries to 18% in Malawi.[2] Preterm labor is a complex phenotype thought to be triggered by several causes including microbial-induced inflammation, maternal stress, uterine distention, decidual haemorrhage and vascular diseases.[3] Complexities in the underlying pathophysiology of the preterm delivery process have been an obstacle to identify effective biomarkers and therapeutics.[4]

Epidemiological studies consistently reported that maternal height is positively associated with child's gestational age at birth, which has been replicated in different populations, ethnic groups and time periods.[5-8] Using Mendelian randomization methods, we demonstrated that this association is likely causal.[9] Building upon the causal association observed in uterine distention experiments in primates,[10] we hypothesized that birth is triggered by an interaction between uterine size and constantly increasing uterine load (fetal growth). In the current study, we test this hypothesis in humans by modeling the extreme of uterine distention in two natural "experiments": twin versus singleton pregnancy and in singletons with large and small fetuses for their gestational age.

For peer review only

METHODS

Study population

We investigated pregnancy records in the Swedish Medical Birth Register of mothers who were born in four Nordic countries: Sweden, Norway, Denmark and Finland. Since 1973, it is compulsory for every delivery unit in Sweden to provide the data to this register.

Only the first pregnancy of every mother was used, only with spontaneous onset of labor and only with live-born children. Due to the absence of indicators for spontaneous or iatrogenic onset of labor before the year 1990, only births from 1990 to 2013 were included. Deliveries initiated by elective caesarean section or starting with prelabor rupture of membranes were excluded. Further exclusions contained self-reported maternal medical conditions (diabetes, chronic hypertension, chronic kidney disease, inflammatory bowel disease, systemic lupus erythematosus), pregnancy complications reported in hospital records (placental disorders, placenta previa, early separation of placenta, antepartum haemorrhage, polyhydramnios, oligohydramnios) and estimated maternal body-mass index <15 or >45 kg/m^2, maternal age <18 or >45 years, higher order multiple births than twins.

Variable definitions

Pregnancies with maternal height values <140 cm and >200 cm were removed (0.01%), as well as height values that differed by more than 10 cm from other height values of the same mother in other pregnancies (0.12%) (The latter procedure took place prior to the exclusion of higher-parity pregnancies).

Gestational age at birth (expressed in days) was estimated by midwives or obstetricians using the second-trimester ultrasonography method and supported by estimated gestational age at birth reported by the delivery unit.

In order to ensure the initial assumption that twin pregnancies manifest larger uterine load

For peer review only - http://bmjopen.bmj.com/site/about/guidelines.xhtml

For peer review only

than singleton pregnancies, we required that birthweight difference of two twins would be no larger than 2 SD of a mean intra-twin differences in a particular 7-day window of gestational age (**Sup Fig 1**). As a result of this filter, 174 (5.7%) twin pregnancies were removed.

The Medical Birth Register does not collect longitudinal data about the fetal growth (i.e., fetal growth curves). However, we used longitudinally derived intrauterine growth curves of healthy Scandinavian pregnancies with term deliveries derived by Marsal et al.[11] in order to convert birthweight, fetal sex and gestational age at birth data into birthweight Z-scores that can be interpreted as being proportional to the fetal growth rate. The newborns with a Z-score larger than 1.5 in the singleton cohort were classified as large for gestational age (LGA), and newborns with a Z-score lower than -1.5 in the singleton cohort were classified as small for gestational age (SGA), **Sup Fig 2**.

Data analysis

We explored how uterine load modulates the effect of maternal height on child's gestational age at birth. We used two models of uterine load: Model 1 with twin versus singleton pregnancies, and Model 2 with LGA versus SGA pregnancies (all singleton). We assumed that at the time of delivery, twins and LGA singletons manifest a larger volumetric uterine load than singletons and SGA singletons, respectively.

In both models, we ran multiple linear regression with child's gestational age at birth (GA) being dependent on continuous maternal height (MH), uterine load (UL) categories, and their interaction, where large uterine load was coded as "1" (twins or LGA) and small uterine load coded as "0" (singletons or SGA):

$$GA = \beta_0 + \beta_1 * MH + \beta_2 * UL + \beta_3 * MH * UL + \varepsilon$$

A simple linear regression was preferred to the more complex ones (using polynomial terms

For peer review only - http://bmjopen.bmj.com/site/about/guidelines.xhtml

and covariates) in order to facilitate interpretability of the interaction patterns. The study population, depleted from risk factors associated with gestational age and height, further justified this choice.

Additionally, we used logistic regression to investigate the same phenomenon in the clinical phenotype - preterm delivery (PTD; birth occurring earlier than 259 days of gestation; standard definition). In this analysis we used extreme dichotomous definitions of maternal stature and uterine load. Short stature was defined by maternal height <161 cm (10th percentile, rounded to integers). In Model 1, high volumetric uterine load was assigned to twin pregnancies, and in Model 2, high volumetric uterine load was assigned to singleton pregnancies with extreme fetal growth, defined by fetus having a birthweight Z-score larger than 2.75 SD. Statistical significance was reported as interaction term P-value.

All statistical analyses were performed with R software for statistical computing (version 3.3.1). The analytical code can be found on github.com/PerinatalLab/MatHeight-GestAge/.

Simulation

We modeled the hypothesized phenomenon in a computer simulation to make sure that the used assumptions were sufficient to produce results similar to the ones generated from the real data. The assumptions can be summarized as follows. A birth concludes gestation due to one of the two types of competing latent events. These two types of latent events are represented by a baseline risk (unmodeled/unknown factors influencing the length of gestation) and the risk imposed due to uterine distention. The baseline-risk events are independent of fetal and maternal measurements, while uterine distention risk is dependent on the ratio between fetal growth rate and uterine size. Child's gestational age in each pregnancy is defined by that latent event, which occurred first.

For peer review only - http://bmjopen.bmj.com/site/about/guidelines.xhtml

Each pregnancy was assigned a randomly generated maternal uterine size and fetal growth rate, following a normal distribution. Maternal height was derived as a correlate of uterine size. As simulation time progressed (i.e., "gestation"), gestational age at birth was recorded for each pregnancy. Generated data was used to test the interaction effect in the same way as described in the Data Analysis. The simulation code can be found in the same Github repository.

Patient involvement

Patients were not involved in setting the research questions or planning the study. Investigators do not know the identity of study participants.

For peer review only

For peer review only - http://bmjopen.bmj.com/site/about/guidelines.xhtml

RESULTS

Our study, investigating uncomplicated first pregnancies of healthy Nordic-born mothers with spontaneous onset of labor, contained 2 846 twin and 527 868 singleton pregnancies. Singleton pregnancies carried 24 286 LGA fetuses and 33 780 SGA fetuses (**Fig 1**). The fractions of excluded pregnancies at each stage are reported in the Sup Table 1.

Gestational age

In Model 1, we investigated the relationship between child's gestational age and maternal height in two categories: women with twin and singleton pregnancies. A clear interaction pattern was detected (**Fig 2, Panel 1**): the differing slopes show that the effect of maternal height on gestational age is modulated by uterine load. In particular, the timing of birth was more sensitive to maternal height in twin pregnancies than in singleton pregnancies. Shorter mothers with twin pregnancies deliver earlier than it would be expected based on the trend in singleton pregnancies and the mean difference between gestational age in singleton and twin pregnancies. The coefficients estimated in multiple linear regression (**Table 1**) show that maternal height has 3 times larger positive linear effect on gestational age in twin pregnancies ($\beta_3+\beta_1$=0.38 days/cm) than it has in singleton pregnancies (β_1=0.13 days/cm). The observed effect-modulating behavior of uterine load (β_3 coefficient) is statistically significant (interaction P=5e-14) and clearly visible in Fig 2.

Table 1. Estimated effect sizes in Model 1 from multiple linear regression. Estimate is expressed in days of gestation. MH - maternal height (cm), UL - uterine load (singletons=0, twins=1). SE - standard error, P - statistical significance.

Coefficient	Estimate	SE	P
β_0 (intercept)	258.2	0.412	<1E-150
β_1 (MH)	0.131	0.002	<1E-150
β_2 (UL)	-69.20	5.619	8E-35
β_3 (MH x UL)	0.252	0.033	5E-14

For peer review only - http://bmjopen.bmj.com/site/about/guidelines.xhtml

In Model 2, we observed similar results: a clear interaction pattern was observed (**Fig 2, Panel 2**), where timing of birth was more sensitive to maternal height in singleton pregnancies with LGA fetuses than singleton pregnancies with SGA fetuses. Shorter mothers with LGA pregnancies deliver earlier than it would be expected based on the trend in SGA pregnancies and the mean difference between gestational age in SGA and LGA pregnancies. The coefficients estimated in multiple linear regression (**Table 2**) show that maternal height has 2 times larger positive linear effect on gestational age in LGA pregnancies ($\beta_3+\beta_1$=0.2 days/cm) than it has in SGA pregnancies (β_1=0.1 days/cm). The observed effect-modulating behavior of volumetric uterine load (β_3 coefficient) is statistically significant (interaction P=1e-11) and clearly visible in Fig 2.

Table 2. Estimated effect sizes in Model 2 from multiple linear regression. Estimate is expressed in days of gestation. MH - maternal height (cm), UL - uterine load (SGA=0, LGA=1). SE - standard error, P - statistical significance.

Coefficient	Estimate	SE	P
β_0 (intercept)	264.6	1.646	<1E-150
β_1 (MH)	0.096	0.0099	5E-22
β_2 (UL)	-20.13	2.598	1E-14
β_3 (MH x UL)	0.105	0.0154	1E-11

Preterm birth

In additional analyses focusing on the clinical phenotype preterm birth, we observed that preterm birth was more prevalent in the risk group of "short mother and high uterine load" than it would be expected without synergistic interaction between the two risk factors "short mother" and "high uterine load".

In Model 1 (**Fig 3, Panel 1**), the odds ratio for preterm birth in the "short mother carrying twins" risk group (Group D, OR=56.2) was larger than it would be expected without interaction between the risk factors "short mother" and "twin pregnancy" (OR=1.42×32.8=46.6). However, the interaction term in the logistic regression was not

For peer review only - http://bmjopen.bmj.com/site/about/guidelines.xhtml

statistically significant (P=0.1).

In Model 2 (**Fig 3, Panel 2**), the odds ratio for preterm birth in the "short mother with extreme fetal growth" risk group (Group D, OR=10.2) was larger than it would be expected without interaction (OR=1.42×2.76=3.92). The corresponding interaction term in the logistic regression was statistically significant (P=0.0005).

Simulation

In a simulation experiment, we tested whether the observed interaction pattern **(Fig 2, Panel 2**) could be qualitatively reproduced, if birth events were indeed to be triggered by fetal size reaching a certain threshold determined by height-correlated uterine size. The observed interaction pattern was replicated in a simulation (**Sup Fig 3**), where birth events were initiated by two competing risks: baseline (unknown environmental and genetic factors) and interaction between fetal growth rate and height-correlated uterine size. Similarly to **Fig 2 (Panel 2)**, LGA pregnancies had a steeper slope than SGA pregnancies.

For peer review only

For peer review only - http://bmjopen.bmj.com/site/about/guidelines.xhtml

DISCUSSION

In this retrospective cohort study we investigated first pregnancies of healthy Nordic-born mothers delivering in Sweden during the period 1990-2013 with spontaneous onset of labor. We found that child's gestational age is more sensitive to maternal height in pregnancies with larger uterine load than in pregnancies with smaller uterine load: the effect of maternal height is significantly larger in twin versus singleton pregnancies, and in singleton pregnancies with fast- vs slow-growing fetus. For example, the duration of twin pregnancies is often shorter than of singleton pregnancies. However, in twin pregnancies, a maternal height increase of 1 cm prolonged pregnancy by 0.38 days; while in singleton pregnancies, a similar increase in maternal height prolonged pregnancy only by 0.13 days. As supported by our simulation, the he observed statistical interaction is likely a manifestation of biomechanical interplay between uterine size and uterine load (fetal size) in time. The labor in uncomplicated spontaneous delivery is likely triggered by uterine distention, which intensifies with the progression of pregnancy and reaches its peak when fetal size approaches the uterine size.

Our results (interaction pattern) support the previous causality claims for maternal height[9] and experimental uterine stretch determining the timing of birth,[10,12,13] and suggests that gestation-imposed uterine stretch also affects birth timing in spontaneous human pregnancies. An alternative explanation could be related to metabolic constraint imposed by maternal energy expenditure and fetal energy demand over gestational time,[14] as twins and LGA singletons require more energy to support their growth and maternal basal metabolism is related to height.[15]

A possible limitation of this study is the assumption that maternal height is a good proxy for uterine size. Similarly, the natural models of uterine distention are not ideal: uterine load groups also differ in other biological aspects,[16] which cannot be fully accounted for. Another potential limitation is that SGA and LGA were defined using birthweight Z-score,

For peer review only - http://bmjopen.bmj.com/site/about/guidelines.xhtml

which was estimated using longitudinally derived intrauterine growth curves of healthy Scandinavian pregnancies with term deliveries,[11] i.e., not ideally representative population.

The biomechanical explanation of timing of birth could benefit genomic and proteomic association studies of preterm birth by narrowing the number of biomarkers and increasing statistical power: our results could support the use of candidate biomarkers from inflammation-mediated pathways, which are excited by uterine distention.[10,17-20]

The future predictive algorithms could take into account fetal growth speed and uterine size while trying to classify pregnancies as being low or high risk for preterm birth.

CONCLUSION

The observed interactions support the hypothesis of uterine distention playing a causal role in determining the timing of birth, at the same time giving support to the causal effect of maternal height, uterine load, uterine size.

ACKNOWLEDGMENTS, COMPETING INTERESTS, FUNDING AND OTHER

Acknowledgements We are grateful to The Swedish National Board of Health and Welfare (*Socialstyrelsen*) who manages the Swedish Medical Birth Register.

Contributors JB and GZ - study design; BJ and VS - data acquisition; JB and JJ - data cleaning and figures; JB and GZ - data analysis and interpretation, KAW - literature search; JB and KAW- writing; BJ, JJ, LJM, VS, GZ - critical revision. All authors approved the final version to be submitted for publication.

Funding This work was supported by Swedish government grants to researchers in the public health sector (ALFGBG-717501, ALFGBG-507701, ALFGBG-426411), The Swedish Research Council (2015-02559), March of Dimes Foundation (21-FY16-121) and the Burroughs Wellcome Fund Preterm Birth Research Grant (10172896).

Competing interests None declared.

Ethics approval The work was approved by the Regional Ethical Review Board in the Western Health Care Region of Sweden (Dnr:968-14).

Provenance and peer review Not commissioned; externally peer reviewed.

For peer review only - http://bmjopen.bmj.com/site/about/guidelines.xhtml

Data sharing statement Researchers can apply for data access by contacting The Swedish National Board of Health and Welfare.

For peer review only

For peer review only - http://bmjopen.bmj.com/site/about/guidelines.xhtml

REFERENCES

1. Liu L, Oza S, Hogan D, et al. Global, regional, and national causes of under-5 mortality in 2000-15: an updated systematic analysis with implications for the Sustainable Development Goals. *Lancet* 2016;388(10063):3027-35. doi: 10.1016/S0140-6736(16)31593-8 [published Online First: 2016/11/15].

2. Blencowe H, Cousens S, Oestergaard MZ, et al. National, regional, and worldwide estimates of preterm birth rates in the year 2010 with time trends since 1990 for selected countries: a systematic analysis and implications. *Lancet* 2012;379(9832):2162-72. doi: 10.1016/S0140-6736(12)60820-4 [published Online First: 2012/06/12].

3. Romero R, Dey SK, Fisher SJ. Preterm labor: one syndrome, many causes. *Science* 2014;345(6198):760-5. doi: 10.1126/science.1251816 [published Online First: 2014/08/16].

4. Iams JD, Romero R, Culhane JF, et al. Primary, secondary, and tertiary interventions to reduce the morbidity and mortality of preterm birth. *Lancet* 2008;371(9607):164-75. doi: 10.1016/S0140-6736(08)60108-7 [published Online First: 2008/01/15].

5. Chan BC, Lao TT. Maternal height and length of gestation: does this impact on preterm labour in Asian women? *Aust N Z J Obstet Gynaecol* 2009;49(4):388-92. doi: 10.1111/j.1479-828X.2009.01006.x [published Online First: 2009/08/22].

6. Dickey RP, Xiong X, Xie Y, et al. Effect of maternal height and weight on risk for preterm singleton and twin births resulting from IVF in the United States, 2008-2010. *Am J Obstet Gynecol* 2013;209(4):349 e1-6. doi: 10.1016/j.ajog.2013.05.052 [published Online First: 2013/06/04].

7. Derraik JG, Lundgren M, Cutfield WS, et al. Maternal Height and Preterm Birth: A Study on 192,432 Swedish Women. *PLoS One* 2016;11(4):e0154304. doi: 10.1371/journal.pone.0154304 [published Online First: 2016/04/23].

8. Morken NH, Kallen K, Jacobsson B. Predicting risk of spontaneous preterm delivery in women with a singleton pregnancy. *Paediatric and perinatal epidemiology* 2014;28(1):11-22. doi: 10.1111/ppe.12087 [published Online First: 2013/10/15].

9. Zhang G, Bacelis J, Lengyel C, et al. Assessing the Causal Relationship of Maternal Height on Birth Size and Gestational Age at Birth: A Mendelian Randomization Analysis. *PLoS medicine* 2015;12(8):e1001865. doi: 10.1371/journal.pmed.1001865 [published Online First: 2015/08/19].

10. Adams Waldorf KM, Singh N, Mohan AR, et al. Uterine overdistention induces preterm labor mediated by inflammation: observations in pregnant women and nonhuman primates. *Am J Obstet Gynecol* 2015;213(6):830 e1-30 e19. doi: 10.1016/j.ajog.2015.08.028 [published Online First: 2015/08/19].

11. Marsal K, Persson PH, Larsen T, et al. Intrauterine growth curves based on ultrasonically estimated foetal weights. *Acta Paediatr* 1996;85(7):843-8. [published Online First: 1996/07/01].

12. Manabe Y, Mori T, Yoshida Y. Decidual morphology and F prostaglandin in amniotic fluid in stretch-induced abortion. *Obstet Gynecol* 1984;64(5):661-5. [published Online First: 1984/11/01].

For peer review only - http://bmjopen.bmj.com/site/about/guidelines.xhtml

13. Manabe Y, Manabe A, Takahashi A. Effect of indomethacin on stretch-induced uterine activity in the post-partum. *Prostaglandins* 1983;25(5):653-9. [published Online First: 1983/05/01].

14. Dunsworth HM, Warrener AG, Deacon T, et al. Metabolic hypothesis for human altriciality. *Proc Natl Acad Sci U S A* 2012;109(38):15212-6. doi: 10.1073/pnas.1205282109 [published Online First: 2012/08/31].

15. Harris JA, Benedict FG. A Biometric Study of Human Basal Metabolism. *Proc Natl Acad Sci U S A* 1918;4(12):370-3. [published Online First: 1918/12/01].

16. Hancock SN, Oliver MH, McLean C, et al. Size at birth and adult fat mass in twin sheep are determined in early gestation. *J Physiol* 2012;590(5):1273-85. doi: 10.1113/jphysiol.2011.220699

17. Loudon JA, Sooranna SR, Bennett PR, et al. Mechanical stretch of human uterine smooth muscle cells increases IL-8 mRNA expression and peptide synthesis. *Mol Hum Reprod* 2004;10(12):895-9. doi: 10.1093/molehr/gah112

18. Sooranna SR, Engineer N, Loudon JA, et al. The mitogen-activated protein kinase dependent expression of prostaglandin H synthase-2 and interleukin-8 messenger ribonucleic acid by myometrial cells: the differential effect of stretch and interleukin-1{beta}. *J Clin Endocrinol Metab* 2005;90(6):3517-27. doi: 10.1210/jc.2004-1390

19. Sooranna SR, Lee Y, Kim LU, et al. Mechanical stretch activates type 2 cyclooxygenase via activator protein-1 transcription factor in human myometrial cells. *Mol Hum Reprod* 2004;10(2):109-13.

20. Terzidou V, Sooranna SR, Kim LU, et al. Mechanical stretch up-regulates the human oxytocin receptor in primary human uterine myocytes. *J Clin Endocrinol Metab* 2005;90(1):237-46. doi: 10.1210/jc.2004-0277

FIGURE LEGENDS

Fig 1. Flowchart for selection of analyzed pregnancies. AGA - child's birthweight is appropriate for gestational age; LGA and SGA - birthweight is large and small for gestational age, respectively. BMI - Body-mass index. LMP - last menstrual period. Two grey boxes represent the analysis in Model 1, two yellow boxes represent analysis in Model 2.

Fig 2. Panel 1) Interaction pattern in Model 1, where the uterine load is modeled as twin vs singleton pregnancies. **Panel 2)** Interaction pattern in Model 2, where uterine load is modeled as too large vs too small birthweight for gestational age in singleton pregnancies (LGA and SGA, respectively). Observations are grouped based on maternal height bins. CI - confidence interval. N - count of observations used to estimate mean. The interaction effect of maternal height and uterine load on gestational duration is demonstrated by the different slopes of the two regression lines calculated in the two groups with a different uterine load.

For peer review only - http://bmjopen.bmj.com/site/about/guidelines.xhtml

Fig 3. Preterm birth in short mothers with: **Panel 1)** twin pregnancies, **Panel 2)** extreme fetal growth. Short stature is defined as <161cm. Preterm birth is defined as delivery before 259 days of gestation. Extreme fetal growth is defined as >2.75 birthweight Z-score (adjusted for gestational age). The grey angular lines denote 95% confidence interval for the expected odds ratio in stratum D, estimated as the product of odds ratios in strata B and C with the assumption of no interaction. In Panel 1, the odds ratio in stratum D is larger than expected due to the interaction between maternal height and fetal growth (interaction P=0.1, logistic regression). In Panel 2, the odds ratio in stratum D is significantly larger than expected due to the interaction between maternal height and fetal growth (interaction P<0.0005, logistic regression).

For peer review only

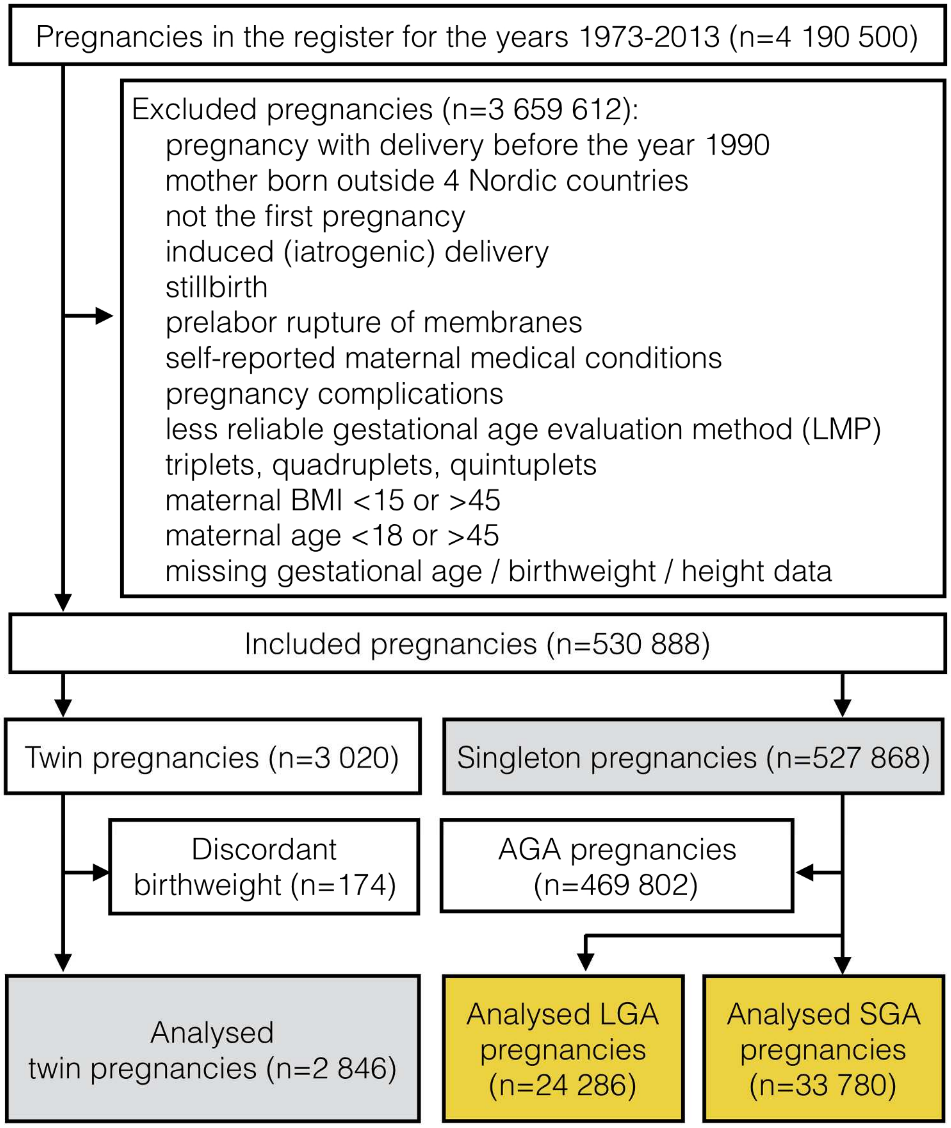

Figure 1. Flowchart for selection of analyzed pregnancies. AGA - child's birthweight is appropriate for gestational age; LGA and SGA - birthweight is large and small for gestational age, respectively. BMI - Body-mass index. LMP - last menstrual period. Two grey boxes represent the analysis in Model 1, two yellow boxes represent analysis in Model 2.

188x220mm (300 x 300 DPI)

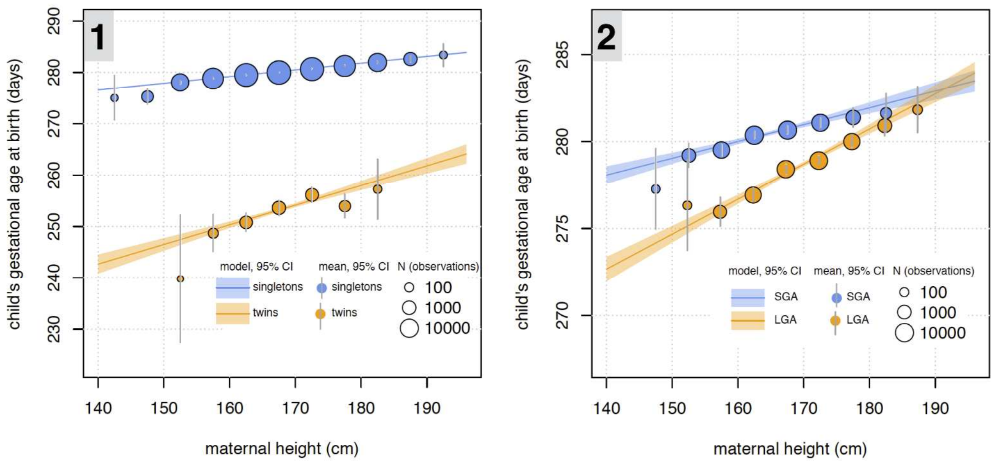

Figure 2. Panel 1) Interaction pattern in Model 1, where the uterine load is modeled as twin vs singleton pregnancies. **Panel 2)** Interaction pattern in Model 2, where uterine load is modeled as too large vs too small birthweight for gestational age in singleton pregnancies (LGA and SGA, respectively). Observations are grouped based on maternal height bins. CI - confidence interval. N - count of observations used to estimate mean. The interaction effect of maternal height and uterine load on gestational duration is demonstrated by the different slopes of the two regression lines calculated in the two groups with a different uterine load.

354x167mm (300 x 300 DPI)

For peer review only

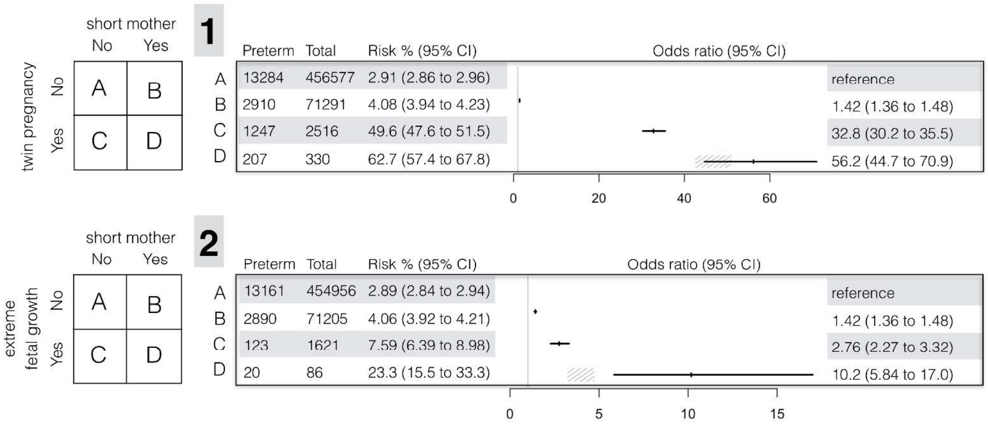

Figure 3. Preterm birth in short mothers with: **Panel 1)** twin pregnancies, **Panel 2)** extreme fetal growth. Short stature is defined as <161cm. Preterm birth is defined as delivery before 259 days of gestation. Extreme fetal growth is defined as >2.75 birthweight Z-score (adjusted for gestational age). The grey angular lines denote 95% confidence interval for the expected odds ratio in stratum D, estimated as the product of odds ratios in strata B and C with the assumption of no interaction. In Panel 1, the odds ratio in stratum D is larger than expected due to the interaction between maternal height and fetal growth (interaction P=0.1, logistic regression). In Panel 2, the odds ratio in stratum D is significantly larger than expected due to the interaction between maternal height and fetal growth (interaction P<0.0005, logistic regression).

331x146mm (300 x 300 DPI)

For peer review only - http://bmjopen.bmj.com/site/about/guidelines.xhtml

Supplementary File

"Uterine distention as a factor in birth timing: retrospective nationwide cohort study in Sweden". Bacelis J. et al. 2018.

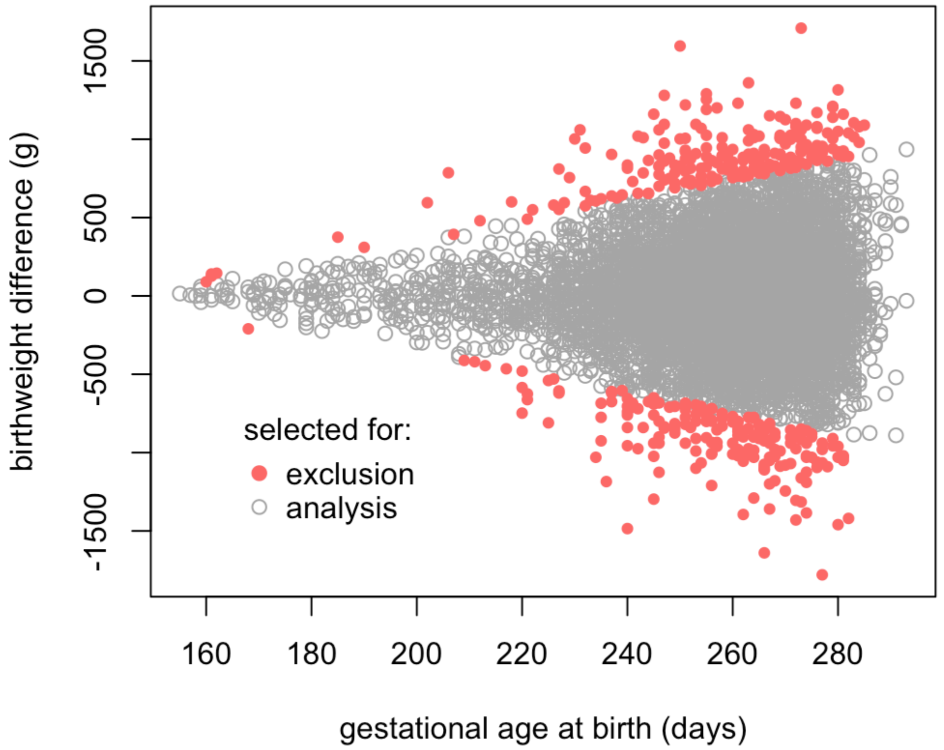

Sup Fig 1 I Selection of twin pregnancies. Pregnancies were excluded if birthweight of two twins differed by more than 2 SD of all intra-twin differences in that gestational age window. Pregnancies with thus discordant twin birthweights (n=174) were excluded from analyses.

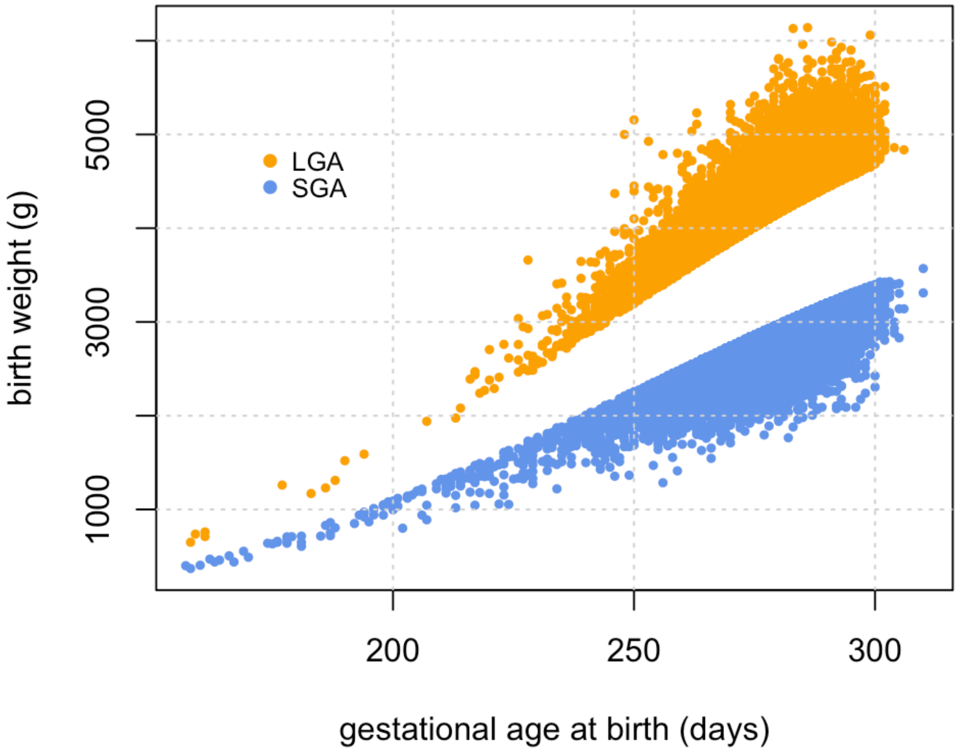

Sup Fig 2 I Classifying singleton births as small (SGA) or large (LGA) for gestational age using birthweight Z-score <-1.5 and >1.5, respectively. Birthweight Z-score was estimated from longitudinally derived intra-uterine growth curves in healthy term pregnancies using polynomial regression equation from Marsal et al., which takes into account child's sex, birthweight, and ultrasound-determined gestational age at birth.

For peer review only - http://bmjopen.bmj.com/site/about/guidelines.xhtml

Supplementary File

"Uterine distention as a factor in birth timing: retrospective nationwide cohort study in Sweden". Bacelis J. et al. 2018.

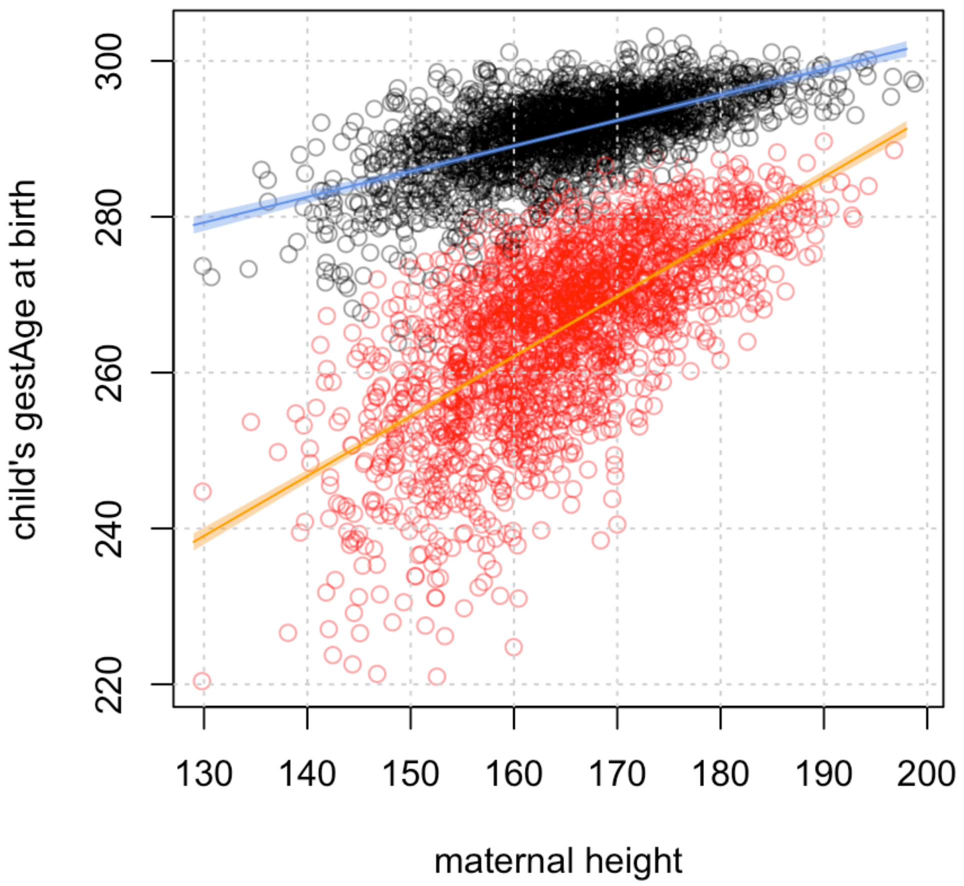

Sup Fig 3 I Simulation of the interaction effect. Gestational age at birth is determined by one of the two competing birth risks. The baseline risk follows a random Gompertz distribution and is independent of any other modeled parameters. The uterine distention risk also follows a Gompertz distribution but is dependent on the ratio between fetal growth rate and uterine size. Each pregnancy has two phantom birth dates, one determined by the baseline risk and one determined by the uterine distention risk. The recorded birth date is the one that occurred first in time (i.e., some of the birth events were triggered by the fetal size reaching the uterine size capacity, and some - by unknown factors). Fetal size at any day of pregnancy is determined by randomly assigned fetal linear growth rate, maternal height is correlated to randomly assigned maternal uterine size. Pregnancies with slow fetal growth are depicted in black circles and blue regression line (with 95% confidence interval); pregnancies with fast fetal growth are depicted in red circles and orange regression line (with 95% confidence interval).

Supplementary File

"Uterine distention as a factor in birth timing: retrospective nationwide cohort study in Sweden". Bacelis J. et al. 2018.

Sup Table 1 | Summary statistics of applied exclusions. This table supplements the flowchart in Figure 1, where it is equivalent to the block named "Excluded pregnancies".

order	exclusion-filter name	remaining rows	rows removed	fraction removed
**1**	maternal ID is missing	4185196	5304	0,13 %
**2**	duplicated maternal ID - child ID combination	4185184	12	0,0003 %
**3**	mother born (or national) outside of 4 Nordic countries	3625825	559359	13 %
**4**	missing, unreliable, <140, >200 maternal height	2616038	1009787	27,8 %
**5**	maternal BMI <15 or >45	2598666	17372	0,66 %
**6**	stillbirth	2587048	11618	0,45 %
**7**	missing gestational age at birth data	2584054	2994	0,12 %
**8**	less reliable gestational age evaluation method (LMP)	1834910	749144	29,0 %
**9**	induced (iatrogenic) delivery (or pregnancy before 1990)	1295490	539420	29,4 %
**10**	prelabor rupture of membranes (PROM)	1276244	19246	1,49 %
**11**	current Caesarean section	1273451	2793	0,22 %
**12**	previous Caesarean section	1206658	66793	5,25 %
**13**	self-reported maternal medical conditions	1188517	18141	1,50 %
**14**	pregnancy complications	1167804	20713	1,74 %
**15**	collapse rows to pregnancies (affects twins, triplets etc.)	1160063	7741	0,66 %
**16**	unclear twin/singleton assignment, triplets etc.	1159635	428	0,037 %
**17**	discordant maternal data in twin entries	1159542	93	0,008 %
**18**	birthweight is missing	1157760	1782	0,15 %
**19**	not the first pregnancy	535300	622460	53,8 %
**20**	maternal age <18 or >45	530888	4412	0,82 %

The authors would like to caution the reader in interpreting the "fraction removed" column: the applied filters overlap (i.e., are correlated), thus the fraction of removed pregnancies due to each filter is highly dependent on the order of applied filters and might not agree well with proportion of certain type of pregnancies estimated in other studies.

STROBE Statement—checklist of items that should be included in reports of observational studies

	Item No.	Recommendation	Page No.	Relevant text from manuscript
**Title and abstract**	1	(*a*) Indicate the study's design with a commonly used term in the title or the abstract	2	"Retrospective cohort study.."
		(*b*) Provide in the abstract an informative and balanced summary of what was done and what was found	2	"Statistical interaction between maternal height and uterine load categories.."
**Introduction**				
Background/rationale	2	Explain the scientific background and rationale for the investigation being reported	3	"..preterm delivery is the leading cause of childhood mortality.."
Objectives	3	State specific objectives, including any prespecified hypotheses	4	"..we hypothesed that birth is triggered by an interaction between uterine size and constantly increasing uterine load.."
**Methods**				
Study design	4	Present key elements of study design early in the paper	5	[pregnancies in Sweden of] "..mothers who were born in four Nordic countries: Sweden, Norway, Denmark and Finland."
Setting	5	Describe the setting, locations, and relevant dates, including periods of recruitment, exposure, follow-up, and data collection	5	"..Swedish Medical Birth Register.." "..1990 to 2013.." "..the first pregnancy of every mother was used.."
Participants	6	(*a*) *Cohort study*—Give the eligibility criteria, and the sources and methods of selection of participants. Describe methods of follow-up *Case-control study*—Give the eligibility criteria, and the sources and methods of case	5	"Deliveries initiated by elective caesarean section or starting with prelabor rupture of

For peer review only - http://bmjopen.bmj.com/site/about/guidelines.xhtml

		ascertainment and control selection. Give the rationale for the choice of cases and controls<br>*Cross-sectional study*—Give the eligibility criteria, and the sources and methods of selection of participants		membranes were removed. Further exclusions contained self-reported maternal medical conditions [...], pregnancy complications.."
		(*b*) *Cohort study*—For matched studies, give matching criteria and number of exposed and unexposed<br>*Case-control study*—For matched studies, give matching criteria and the number of controls per case		
Variables	7	Clearly define all outcomes, exposures, predictors, potential confounders, and effect modifiers. Give diagnostic criteria, if applicable	6<br><br>6	"..child's gestational age at birth (GA) being dependent on continuous maternal height (MH), uterine load (UL) categories.."<br>"..uterine load: [1] twin versus singleton pregnancies, and [2] LGA versus SGA pregnancies.."
Data sources/ measurement	8*	For each variable of interest, give sources of data and details of methods of assessment (measurement). Describe comparability of assessment methods if there is more than one group	*5,6*	"Maternal height.."<br>"Gestational age.."<br>"Twin pregnancies.."<br>"Fetal Growth.."
Bias	9	Describe any efforts to address potential sources of bias	6<br><br>7	"We used two [very different] models of uterine load.."<br>"..computer simulation [was used] to make sure that the used assumptions were sufficient.."
Study size	10	Explain how the study size was arrived at	5	Section "Study Population" and below

Continued on next page

For peer review only - http://bmjopen.bmj.com/site/about/guidelines.xhtml

Quantitative variables	11	Explain how quantitative variables were handled in the analyses. If applicable, describe which groupings were chosen and why	7	"..continuous maternal height (MH), uterine load (UL) categories.." and formula below
Statistical methods	12	(*a*) Describe all statistical methods, including those used to control for confounding	6<br>7	"multiple linear regression.."<br>"..logictic regression.."
		(*b*) Describe any methods used to examine subgroups and interactions	7	the whole page
		(*c*) Explain how missing data were addressed	Fig1	excluded pregnancies with "missing gestational age / birthweight / height data"
		(*d*) *Cohort study*—If applicable, explain how loss to follow-up was addressed<br>*Case-control study*—If applicable, explain how matching of cases and controls was addressed<br>*Cross-sectional study*—If applicable, describe analytical methods taking account of sampling strategy		not applicable
		(*e*) Describe any sensitivity analyses	12	"Sensitivity analyses showed.."
**Results**				
Participants	13*	(a) Report numbers of individuals at each stage of study—eg numbers potentially eligible, examined for eligibility, confirmed eligible, included in the study, completing follow-up, and analysed	Fig1	
		(b) Give reasons for non-participation at each stage	Fig1	
		(c) Consider use of a flow diagram	Fig1	
Descriptive data	14*	(a) Give characteristics of study participants (eg demographic, clinical, social) and information on exposures and potential confounders	5	"mothers who were born in four Nordic countries: Sweden, Norway, Denmark and Finland"<br>"Further exclusions contained.."
		(b) Indicate number of participants with missing data for each variable of interest	Fig1	[no individuals with missing data]
		(c) *Cohort study*—Summarise follow-up time (eg, average and total amount)		not aplicable
Outcome data	15*	*Cohort study*—Report numbers of outcome events or summary measures over time		not aplicable (our outcome is interaction three variables)
		Case-control study—Report numbers in each exposure category, or summary measures of exposure	Fig3	somewhat applicable
		Cross-sectional study—Report numbers of outcome events or summary measures		
Main results	16	(*a*) Give unadjusted estimates and, if applicable, confounder-adjusted estimates and their precision (eg, 95% confidence interval). Make clear which confounders were adjusted for and why they were		Not applicable. Confounder adjustment would be too

For peer review only - http://bmjopen.bmj.com/site/about/guidelines.xhtml

	included		complicated to interpret in this particular study investigating interaction effect. We used different approach (two different models of phenomenon and simulation) to address this problem.
	(*b*) Report category boundaries when continuous variables were categorized	6	"The newborns with a Z-score larger than 1.5 in the singleton cohort were classified as large for gestational age (LGA), and newborns with a Z-score lower than -1.5 in the singleton cohort were classified as small for gestational age (SGA).."
	(*c*) If relevant, consider translating estimates of relative risk into absolute risk for a meaningful time period		not relevant

Continued on next page

For peer review only - http://bmjopen.bmj.com/site/about/guidelines.xhtml

Other analyses	17	Report other analyses done—eg analyses of subgroups and interactions, and sensitivity analyses	12	"Sensitivity analyses showed.."
**Discussion**				
Key results	18	Summarise key results with reference to study objectives	12	"..the observed statistical interaction is likely a manifestation of biomechanical interplay between uterine size and uterine load (fetal size) in time.."
Limitations	19	Discuss limitations of the study, taking into account sources of potential bias or imprecision. Discuss both direction and magnitude of any potential bias	12<br>12<br>3	"..An alternative explanation.."<br>"..not when parity was higher.."<br>"..possible limitation of this study.."
Interpretation	20	Give a cautious overall interpretation of results considering objectives, limitations, multiplicity of analyses, results from similar studies, and other relevant evidence	13	"The observed interactions support the hypothesis .."
Generalisability	21	Discuss the generalisability (external validity) of the study results	12	"..not when parity was higher.."
**Other information**				
Funding	22	Give the source of funding and the role of the funders for the present study and, if applicable, for the original study on which the present article is based	13	"Funding.."

*Give information separately for cases and controls in case-control studies and, if applicable, for exposed and unexposed groups in cohort and cross-sectional studies.

Note: An Explanation and Elaboration article discusses each checklist item and gives methodological background and published examples of transparent reporting. The STROBE checklist is best used in conjunction with this article (freely available on the Web sites of PLoS Medicine at http://www.plosmedicine.org/, Annals of Internal Medicine at http://www.annals.org/, and Epidemiology at http://www.epidem.com/). Information on the STROBE Initiative is available at www.strobe-statement.org.

For peer review only - http://bmjopen.bmj.com/site/about/guidelines.xhtml

BMJ Open

Uterine distention as a factor in birth timing: retrospective nationwide cohort study in Sweden

Journal:	*BMJ Open*
Manuscript ID	bmjopen-2018-022929.R2
Article Type:	Research
Date Submitted by the Author:	02-Oct-2018

Complete List of Authors:	Bacelis, Jonas; Sahlgrenska University Hospital Östra, Obstetrics and Gynecology<br>Juodakis, Julius; University of Gothenburg, Sahlgrenska Academy, Institute of Clinical Sciences, dept. Obstetrics and Gynecology<br>Adams Waldorf, Kristina; University of Washington, Obstetrics and Gynecology; Sahlgrenska University Hospital Östra, Obstetrics and Gynecology<br>Sengpiel, Verena; Sahlgrenska University Hospital Östra, Obstetrics and Gynecology<br>Muglia, Louis; Cincinnati Children's Hospital Medical Center, Human Genetics Division; Cincinnati Children's Hospital Medical Center, Center for Prevention of Preterm Birth<br>Zhang, Ge; Cincinnati Children's Hospital Medical Center, Human Genetics Division; Cincinnati Children's Hospital Medical Center, Center for Prevention of Preterm Birth<br>Jacobsson, Bo; Sahlgrenska University Hospital Östra, Obstetrics and Gynecology; Nasjonalt folkehelseinstitutt, Genetics and Bioinformatics

**Primary Subject Heading**:	Obstetrics and gynaecology
Secondary Subject Heading:	Epidemiology
Keywords:	Gestational age at birth, Large for gestational age, Small for gestational age, Preterm delivery, Twins, Uterine distention

SCHOLARONE™
Manuscripts

TITLE PAGE

Title:

Uterine distention as a factor in birth timing: retrospective nationwide cohort study in Sweden

Corresponding author:

Jonas Bacelis

Diagnosvägen 15, 416 50, Gothenburg, Sweden

jonas.bacelis@gu.se, +46769259045

All co-authors:

Bacelis J[1$], Juodakis J[2], Adams Waldorf KM[1,3,4], Sengpiel V[1], Muglia LJ[5,6], Zhang G[5,6*], Jacobsson B[1,7*]

[1] Department of Obstetrics and Gynecology, Sahlgrenska University Hospital Östra, Gothenburg, Sweden

[2] Department of Obstetrics and Gynecology, Institute of Clinical Sciences, Sahlgrenska Academy, University of Gothenburg, Gothenburg, Sweden

[3] Department of Obstetrics and Gynecology, University of Washington, Seattle, Washington, USA

[4] Department of Global Health, University of Washington, Seattle, Washington, USA

[5] Human Genetics Division, Cincinnati Children's Hospital Medical Center, Cincinnati, Ohio, USA

[6] Center for Prevention of Preterm Birth, Perinatal Institute, Cincinnati Children's Hospital Medical Center and March of Dimes Prematurity Research Center Ohio Collaborative, Cincinnati, Ohio, USA

[7] Department of Genetics and Bioinformatics, Area of Health Data and Digitalisation, Norwegian Institute of Public Health, Oslo, Norway

[$] Corresponding author, [*] Co-senior authors

Word count:

2100 words

For peer review only

For peer review only - http://bmjopen.bmj.com/site/about/guidelines.xhtml

ABSTRACT

Objectives To determine whether uterine distention is associated with human pregnancy duration in a non-invasive observational setting.

Design Retrospective cohort study modeling uterine distention by interaction between maternal height and uterine load.

Setting The study is based on the 1990-2013 population data from all delivery units in Sweden.

Participants Uncomplicated first pregnancies of healthy Nordic-born mothers with spontaneous onset of labor. Pregnancies were classified as twin (n=2,846) or singleton (n=527,868). Singleton pregnancies were further classified as carrying a large for gestational age fetus (LGA, n=24,286) or small for gestational age fetus (SGA, n=33,780).

Outcome measures Statistical interaction between maternal height and uterine load categories (twin vs singleton pregnancies, and LGA vs SGA singleton pregnancies), where the outcome is pregnancy duration.

Results In all models, statistically significant interaction was found. Mothers carrying twins had 2.9 times larger positive linear effect of maternal height on gestational age than mothers carrying singletons (interaction p=5e-14). Similarly, the effect of maternal height was strongly modulated by the fetal growth rate in singleton pregnancies: the effect size of maternal height on gestational age in LGA pregnancies was 2.1 times larger than that in SGA pregnancies (interaction p<1e-11). Preterm birth odds ratio was 1.4 when the mother was short, and 2.8 when the fetus was extremely large for its gestational age; however, when both risk factors were present together, the odds ratio for preterm birth was larger than expected, 10.2 (interaction p<0.0005).

Conclusions Across all classes, maternal height was significantly associated with child's gestational age at birth. Interestingly, in short-statured women, spontaneous delivery occurred much earlier than expected when the uterine load was large (twins, LGA). The interaction between maternal height, uterine load size and gestational age at birth strongly suggests the effect of uterine distention imposed by fetal growth on birth timing.

Keywords Gestational age at birth, Large for gestational age, Small for gestational age, Preterm delivery, Preterm birth, Twins, Uterine distention. (**Additional keywords:** Parturition, Pregnancy, Causality, Fetal growth rate, Gestational length, Interaction, Labor, Uterine load.)

For peer review only - http://bmjopen.bmj.com/site/about/guidelines.xhtml

STRENGTHS AND LIMITATIONS OF THE STUDY

- This is the first study to examine the effect of pregnancy-related uterine distention in a medical register data (i.e., non-invasively).
- We used uncomplicated pregnancies from a large, healthy, homogeneous population depleted from risk factors associated with gestational age and adult height.
- To minimize confounding, uterine distention was modeled by two natural phenomena: twin pregnancies and singleton fetuses with very rapid growth.
- The study has implications to the design of future epidemiological, genetic, and proteomic preterm birth studies.
- A possible limitation of this study is the assumption that maternal height is a good proxy for uterine size.

For peer review only

For peer review only - http://bmjopen.bmj.com/site/about/guidelines.xhtml

INTRODUCTION

Preterm birth remains the leading cause of adverse outcomes of pregnancy. Globally preterm delivery is the leading cause of childhood mortality (under-five).[1] Indeed, the long-term health of the child is dependent to a great extent on the length of time spent in the mother's womb as a fetus. Worldwide, preterm delivery rates range from about 5% in some Northern European countries to 18% in Malawi.[2] Preterm labor is a complex phenotype thought to be triggered by several causes including microbial-induced inflammation, maternal stress, uterine distention, decidual haemorrhage and vascular diseases.[3] Complexities in the underlying pathophysiology of the preterm delivery process have been an obstacle to identify effective biomarkers and therapeutics.[4]

Epidemiological studies consistently reported that maternal height is positively associated with child's gestational age at birth, which has been replicated in different populations, ethnic groups and time periods.[5-8] Using Mendelian randomization methods, we demonstrated that this association is likely causal.[9] Building upon the causal association observed in uterine distention experiments in primates,[10] we hypothesized that birth is triggered by an interaction between uterine size and constantly increasing uterine load (fetal growth). In the current study, we test this hypothesis in humans by modeling the extreme of uterine distention in two natural "experiments": twin versus singleton pregnancy and in singletons with large and small fetuses for their gestational age.

For peer review only

METHODS

Study population

We investigated pregnancy records in the Swedish Medical Birth Register of mothers who were born in four Nordic countries: Sweden, Norway, Denmark and Finland. Since 1973, it is compulsory for every delivery unit in Sweden to provide the data to this register.

Only the first pregnancy of every mother was used, only with spontaneous onset of labor and only with live-born children. Due to the absence of indicators for spontaneous or iatrogenic onset of labor before the year 1990, only births from 1990 to 2013 were included. Deliveries initiated by elective caesarean section or starting with prelabor rupture of membranes were excluded. Further exclusions contained self-reported maternal medical conditions (diabetes, chronic hypertension, chronic kidney disease, inflammatory bowel disease, systemic lupus erythematosus), pregnancy complications reported in hospital records (placental disorders, placenta previa, early separation of placenta, antepartum haemorrhage, polyhydramnios, oligohydramnios) and estimated maternal body-mass index <15 or >45 kg/m^2, maternal age <18 or >45 years, higher order multiple births than twins.

Variable definitions

Pregnancies with maternal height values <140 cm and >200 cm were removed (0.01%), as well as height values that differed by more than 10 cm from other height values of the same mother in other pregnancies (0.12%) (The latter procedure took place prior to the exclusion of higher-parity pregnancies).

Gestational age at birth (expressed in days) was estimated by midwives or obstetricians using the second-trimester ultrasonography method and supported by estimated gestational age at birth reported by the delivery unit.

In order to ensure the initial assumption that twin pregnancies manifest larger uterine load

For peer review only - http://bmjopen.bmj.com/site/about/guidelines.xhtml

than singleton pregnancies, we required that birth weight difference of two twins would be no larger than 2 SD of a mean intra-twin differences in a particular 7-day window of gestational age (**Sup Fig 1**). As a result of this filter, 174 (5.7%) twin pregnancies were removed.

The Medical Birth Register does not collect longitudinal data about the fetal growth (i.e., fetal growth curves). However, we used longitudinally derived intrauterine growth curves of healthy Scandinavian pregnancies with term deliveries derived by Marsal et al.[11] in order to convert birth weight, fetal sex and gestational age at birth data into birth weight Z-scores that can be interpreted as being proportional to the fetal growth rate. The newborns with a Z-score larger than 1.5 in the singleton cohort were classified as large for gestational age (LGA), and newborns with a Z-score lower than -1.5 in the singleton cohort were classified as small for gestational age (SGA), **Sup Fig 2**.

Data analysis

We explored how uterine load modulates the effect of maternal height on child's gestational age at birth. We used two models of uterine load: Model 1 with twin versus singleton pregnancies, and Model 2 with LGA versus SGA pregnancies (all singleton). We assumed that at the time of delivery, twins and LGA singletons manifest a larger volumetric uterine load than singletons and SGA singletons, respectively.

In both models, we ran multiple linear regression with child's gestational age at birth (GA) being dependent on continuous maternal height (MH), uterine load (UL) categories, and their interaction, where large uterine load was coded as "1" (twins or LGA) and small uterine load coded as "0" (singletons or SGA):

$$GA = \beta_0 + \beta_1 * MH + \beta_2 * UL + \beta_3 * MH * UL + \varepsilon$$

A simple linear regression was preferred to more complex ones (using polynomial terms and

For peer review only - http://bmjopen.bmj.com/site/about/guidelines.xhtml

covariates) in order to facilitate interpretability of the interaction patterns. The study population, depleted from risk factors associated with gestational age and height, further justified this choice.

Additionally, we used logistic regression to investigate the same phenomenon in the clinical phenotype - preterm delivery (PTD; birth occurring earlier than 259 days of gestation; standard definition). In this analysis we used extreme dichotomous definitions of maternal stature and uterine load. Short stature was defined by maternal height <161 cm (10th percentile, rounded to integers). In Model 1, high volumetric uterine load was assigned to twin pregnancies, and in Model 2, high volumetric uterine load was assigned to singleton pregnancies with extreme fetal growth, defined by fetus having a birth weight Z-score larger than 2.75 SD. Statistical significance was reported as interaction term P-value.

All statistical analyses were performed with R software for statistical computing (version 3.3.1). The analytical code can be found on github.com/PerinatalLab/MatHeight-GestAge/.

Patient involvement

Patients were not involved in setting the research questions or planning the study. Investigators do not know the identity of study participants.

For peer review only - http://bmjopen.bmj.com/site/about/guidelines.xhtml

RESULTS

Our study, investigating uncomplicated first pregnancies of healthy Nordic-born mothers with spontaneous onset of labor, contained 2 846 twin and 527 868 singleton pregnancies. Singleton pregnancies carried 24 286 LGA fetuses and 33 780 SGA fetuses (**Fig 1**). The fractions of excluded pregnancies at each stage are reported in the Sup Table 1.

Gestational age

In Model 1, we investigated the relationship between child's gestational age and maternal height in two categories: women with twin and singleton pregnancies. A clear interaction pattern was detected (**Fig 2, Panel 1**): the differing slopes show that the effect of maternal height on gestational age is modulated by uterine load. In particular, the timing of birth was more sensitive to maternal height in twin pregnancies than in singleton pregnancies. Shorter mothers with twin pregnancies deliver earlier than it would be expected based on the trend in singleton pregnancies and the mean difference between gestational age in singleton and twin pregnancies. The coefficients estimated in multiple linear regression (**Table 1**) show that maternal height has 3 times larger positive linear effect on gestational age in twin pregnancies ($\beta_3+\beta_1$=0.38 days/cm) than it has in singleton pregnancies (β_1=0.13 days/cm). The observed effect-modulating behavior of uterine load (β_3 coefficient) is statistically significant (interaction P=5e-14) and clearly visible in Fig 2.

Table 1. Estimated effect sizes in Model 1 from multiple linear regression. Estimate is expressed in days of gestation. MH - maternal height (cm), UL - uterine load (singletons=0, twins=1). SE - standard error, P - statistical significance.

Coefficient	Estimate	SE	P
β_0 (intercept)	258.2	0.412	<1E-150
β_1 (MH)	0.131	0.002	<1E-150
β_2 (UL)	-69.20	5.619	8E-35
β_3 (MH x UL)	0.252	0.033	5E-14

For peer review only - http://bmjopen.bmj.com/site/about/guidelines.xhtml

In Model 2, we observed similar results: a clear interaction pattern was observed (**Fig 2, Panel 2**), where timing of birth was more sensitive to maternal height in singleton pregnancies with LGA fetuses than singleton pregnancies with SGA fetuses. Shorter mothers with LGA pregnancies deliver earlier than it would be expected based on the trend in SGA pregnancies and the mean difference between gestational age in SGA and LGA pregnancies. The coefficients estimated in multiple linear regression (**Table 2**) show that maternal height has 2 times larger positive linear effect on gestational age in LGA pregnancies ($\beta_3+\beta_1=0.2$ days/cm) than it has in SGA pregnancies ($\beta_1=0.1$ days/cm). The observed effect-modulating behavior of volumetric uterine load (β_3 coefficient) is statistically significant (interaction P=1e-11) and clearly visible in Fig 2.

Table 2. Estimated effect sizes in Model 2 from multiple linear regression. Estimate is expressed in days of gestation. MH - maternal height (cm), UL - uterine load (SGA=0, LGA=1). SE - standard error, P - statistical significance.

Coefficient	Estimate	SE	P
β_0 (intercept)	264.6	1.646	<1E-150
β_1 (MH)	0.096	0.0099	5E-22
β_2 (UL)	-20.13	2.598	1E-14
β_3 (MH x UL)	0.105	0.0154	1E-11

Preterm birth

In additional analyses focusing on the clinical phenotype preterm birth, we observed that preterm birth was more prevalent in the risk group of "short mother and high uterine load" than it would be expected without synergistic interaction between the two risk factors "short mother" and "high uterine load".

In Model 1 (**Fig 3, Panel 1**), the odds ratio for preterm birth in the "short mother carrying twins" risk group (Group D, OR=56.2) was larger than it would be expected without interaction between the risk factors "short mother" and "twin pregnancy" (OR=1.42×32.8=46.6). However, the interaction term in the logistic regression was not

For peer review only - http://bmjopen.bmj.com/site/about/guidelines.xhtml

statistically significant (P=0.1).

In Model 2 (**Fig 3, Panel 2**), the odds ratio for preterm birth in the "short mother with extreme fetal growth" risk group (Group D, OR=10.2) was larger than it would be expected without interaction (OR=1.42×2.76=3.92). The corresponding interaction term in the logistic regression was statistically significant (P=0.0005).

Sensitivity analyses

To address the arbitrary nature of the thresholds chosen to define uterine load categories in singleton pregnancies, we ran two sensitivity analyses: (1) SGA and LGA defined by birth weight Z-score in ranges (-2.0, -1.0] and (1.0, 2.0], respectively; (2) using continuous uterine load variable in appropriate for gestational age singletons with birth weight Z-score in range (-2.0, 2.0]. The resulting interaction pattern (**Sup Fig 3 and 4**) was qualitatively identical to the results described in the **Gestational age** section.

DISCUSSION

In this retrospective cohort study we investigated first pregnancies of healthy Nordic-born mothers delivering in Sweden during the period 1990-2013 with spontaneous onset of labor. We found that child's gestational age is more sensitive to maternal height in pregnancies with larger uterine load than in pregnancies with smaller uterine load: the effect of maternal height is significantly larger in twin versus singleton pregnancies, and in singleton pregnancies with fast- vs slow-growing fetus. For example, the duration of twin pregnancies is often shorter than of singleton pregnancies. However, in twin pregnancies, a maternal height increase of 1 cm prolonged pregnancy by 0.38 days; while in singleton pregnancies, a similar increase in maternal height prolonged pregnancy only by 0.13 days. The he observed statistical interaction is likely a manifestation of biomechanical interplay between uterine size and

For peer review only - http://bmjopen.bmj.com/site/about/guidelines.xhtml

uterine load (fetal size) in time. The labor in uncomplicated spontaneous delivery is likely triggered by uterine distention, which intensifies with the progression of pregnancy and reaches its peak when fetal size approaches the uterine size.

Our results (interaction pattern) support the previous causality claims for maternal height[9] and experimental uterine stretch determining the timing of birth,[10,12,13] and suggests that gestation-imposed uterine stretch also affects birth timing in spontaneous human pregnancies. An alternative explanation could be related to metabolic constraint imposed by maternal energy expenditure and fetal energy demand over gestational time,[14] as twins and LGA singletons require more energy to support their growth and maternal basal metabolism is related to height.[15]

A possible limitation of this study is the assumption that maternal height is a good proxy for uterine size. Similarly, the natural models of uterine distention are not ideal: uterine load groups also differ in other biological aspects,[16] which cannot be fully accounted for. Another potential limitation is that SGA and LGA were defined using birth weight Z-score, which was estimated using longitudinally derived intrauterine growth curves of healthy Scandinavian pregnancies with term deliveries,[11] i.e., not ideally representative population.

The biomechanical explanation of timing of birth could benefit genomic and proteomic association studies of preterm birth by narrowing the number of biomarkers and increasing statistical power: our results could support the use of candidate biomarkers from inflammation-mediated pathways, which are excited by uterine distention.[10,17-20]

The future predictive algorithms could take into account fetal growth speed and uterine size while trying to classify pregnancies as being low or high risk for preterm birth.

CONCLUSION

For peer review only - http://bmjopen.bmj.com/site/about/guidelines.xhtml

The observed interactions support the hypothesis of uterine distention playing a causal role in determining the timing of birth, at the same time giving support to the causal effect of maternal height, uterine load, uterine size.

ACKNOWLEDGMENTS, COMPETING INTERESTS, FUNDING AND OTHER

Acknowledgements We are grateful to The Swedish National Board of Health and Welfare (*Socialstyrelsen*) which manages the Swedish Medical Birth Register.

Contributors JB and GZ - study design; BJ and VS - data acquisition; JB and JJ - data cleaning and figures; JB and GZ - data analysis and interpretation, KAW - literature search; JB and KAW- writing; BJ, JJ, LJM, VS, GZ - critical revision. All authors approved the final version to be submitted for publication.

Funding This work was supported by Swedish government grants to researchers in the public health sector (ALFGBG-717501, ALFGBG-507701, ALFGBG-426411), The Swedish Research Council (2015-02559), March of Dimes Foundation (21-FY16-121) and the Burroughs Wellcome Fund Preterm Birth Research Grant (10172896).

Competing interests None declared.

Ethics approval The work was approved by the Regional Ethical Review Board in the Western Health Care Region of Sweden (Dnr:968-14).

Provenance and peer review Not commissioned; externally peer reviewed.

Data sharing statement Researchers can apply for data access by contacting The Swedish National Board of Health and Welfare.

For peer review only

For peer review only - http://bmjopen.bmj.com/site/about/guidelines.xhtml

REFERENCES

1. Liu L, Oza S, Hogan D, et al. Global, regional, and national causes of under-5 mortality in 2000-15: an updated systematic analysis with implications for the Sustainable Development Goals. *Lancet* 2016;388(10063):3027-35. doi: 10.1016/S0140-6736(16)31593-8 [published Online First: 2016/11/15].

2. Blencowe H, Cousens S, Oestergaard MZ, et al. National, regional, and worldwide estimates of preterm birth rates in the year 2010 with time trends since 1990 for selected countries: a systematic analysis and implications. *Lancet* 2012;379(9832):2162-72. doi: 10.1016/S0140-6736(12)60820-4 [published Online First: 2012/06/12].

3. Romero R, Dey SK, Fisher SJ. Preterm labor: one syndrome, many causes. *Science* 2014;345(6198):760-5. doi: 10.1126/science.1251816 [published Online First: 2014/08/16].

4. Iams JD, Romero R, Culhane JF, et al. Primary, secondary, and tertiary interventions to reduce the morbidity and mortality of preterm birth. *Lancet* 2008;371(9607):164-75. doi: 10.1016/S0140-6736(08)60108-7 [published Online First: 2008/01/15].

5. Chan BC, Lao TT. Maternal height and length of gestation: does this impact on preterm labour in Asian women? *Aust N Z J Obstet Gynaecol* 2009;49(4):388-92. doi: 10.1111/j.1479-828X.2009.01006.x [published Online First: 2009/08/22].

6. Dickey RP, Xiong X, Xie Y, et al. Effect of maternal height and weight on risk for preterm singleton and twin births resulting from IVF in the United States, 2008-2010. *Am J Obstet Gynecol* 2013;209(4):349 e1-6. doi: 10.1016/j.ajog.2013.05.052 [published Online First: 2013/06/04].

7. Derraik JG, Lundgren M, Cutfield WS, et al. Maternal Height and Preterm Birth: A Study on 192,432 Swedish Women. *PLoS One* 2016;11(4):e0154304. doi: 10.1371/journal.pone.0154304 [published Online First: 2016/04/23].

8. Morken NH, Kallen K, Jacobsson B. Predicting risk of spontaneous preterm delivery in women with a singleton pregnancy. *Paediatric and perinatal epidemiology* 2014;28(1):11-22. doi: 10.1111/ppe.12087 [published Online First: 2013/10/15].

9. Zhang G, Bacelis J, Lengyel C, et al. Assessing the Causal Relationship of Maternal Height on Birth Size and Gestational Age at Birth: A Mendelian Randomization Analysis. *PLoS medicine* 2015;12(8):e1001865. doi: 10.1371/journal.pmed.1001865 [published Online First: 2015/08/19].

10. Adams Waldorf KM, Singh N, Mohan AR, et al. Uterine overdistention induces preterm labor mediated by inflammation: observations in pregnant women and nonhuman primates. *Am J Obstet Gynecol* 2015;213(6):830 e1-30 e19. doi: 10.1016/j.ajog.2015.08.028 [published Online First: 2015/08/19].

11. Marsal K, Persson PH, Larsen T, et al. Intrauterine growth curves based on ultrasonically estimated foetal weights. *Acta Paediatr* 1996;85(7):843-8. [published Online First: 1996/07/01].

12. Manabe Y, Mori T, Yoshida Y. Decidual morphology and F prostaglandin in amniotic fluid in stretch-induced abortion. *Obstet Gynecol* 1984;64(5):661-5. [published Online First: 1984/11/01].

For peer review only - http://bmjopen.bmj.com/site/about/guidelines.xhtml

13. Manabe Y, Manabe A, Takahashi A. Effect of indomethacin on stretch-induced uterine activity in the post-partum. *Prostaglandins* 1983;25(5):653-9. [published Online First: 1983/05/01].

14. Dunsworth HM, Warrener AG, Deacon T, et al. Metabolic hypothesis for human altriciality. *Proc Natl Acad Sci U S A* 2012;109(38):15212-6. doi: 10.1073/pnas.1205282109 [published Online First: 2012/08/31].

15. Harris JA, Benedict FG. A Biometric Study of Human Basal Metabolism. *Proc Natl Acad Sci U S A* 1918;4(12):370-3. [published Online First: 1918/12/01].

16. Hancock SN, Oliver MH, McLean C, et al. Size at birth and adult fat mass in twin sheep are determined in early gestation. *J Physiol* 2012;590(5):1273-85. doi: 10.1113/jphysiol.2011.220699

17. Loudon JA, Sooranna SR, Bennett PR, et al. Mechanical stretch of human uterine smooth muscle cells increases IL-8 mRNA expression and peptide synthesis. *Mol Hum Reprod* 2004;10(12):895-9. doi: 10.1093/molehr/gah112

18. Sooranna SR, Engineer N, Loudon JA, et al. The mitogen-activated protein kinase dependent expression of prostaglandin H synthase-2 and interleukin-8 messenger ribonucleic acid by myometrial cells: the differential effect of stretch and interleukin-1{beta}. *J Clin Endocrinol Metab* 2005;90(6):3517-27. doi: 10.1210/jc.2004-1390

19. Sooranna SR, Lee Y, Kim LU, et al. Mechanical stretch activates type 2 cyclooxygenase via activator protein-1 transcription factor in human myometrial cells. *Mol Hum Reprod* 2004;10(2):109-13.

20. Terzidou V, Sooranna SR, Kim LU, et al. Mechanical stretch up-regulates the human oxytocin receptor in primary human uterine myocytes. *J Clin Endocrinol Metab* 2005;90(1):237-46. doi: 10.1210/jc.2004-0277

FIGURE LEGENDS

Fig 1. Flowchart for selection of analyzed pregnancies. AGA - child's birth weight is appropriate for gestational age; LGA and SGA - birth weight is large and small for gestational age, respectively. BMI - Body-mass index. LMP - last menstrual period. Two grey boxes represent the analysis in Model 1, two yellow boxes represent analysis in Model 2.

Fig 2. Panel 1) Interaction pattern in Model 1, where the uterine load is modeled as twin vs singleton pregnancies. **Panel 2)** Interaction pattern in Model 2, where uterine load is modeled as too large vs too small birth weight for gestational age in singleton pregnancies (LGA and SGA, respectively). Observations are grouped based on maternal height bins. CI - confidence interval. N - count of observations used to estimate mean. The interaction effect of maternal height and uterine load on gestational duration is demonstrated by the different slopes of the two regression lines calculated in the two groups with a different uterine load.

For peer review only - http://bmjopen.bmj.com/site/about/guidelines.xhtml

Fig 3. Preterm birth in short mothers with: **Panel 1)** twin pregnancies, **Panel 2)** extreme fetal growth. Short stature is defined as <161cm. Preterm birth is defined as delivery before 259 days of gestation. Extreme fetal growth is defined as >2.75 birth weight Z-score (adjusted for gestational age). The grey angular lines denote 95% confidence interval for the expected odds ratio in stratum D, estimated as the product of odds ratios in strata B and C with the assumption of no interaction. In Panel 1, the odds ratio in stratum D is larger than expected due to the interaction between maternal height and fetal growth (interaction $P=0.1$, logistic regression). In Panel 2, the odds ratio in stratum D is significantly larger than expected due to the interaction between maternal height and fetal growth (interaction $P<0.0005$, logistic regression).

For peer review only

For peer review only - http://bmjopen.bmj.com/site/about/guidelines.xhtml

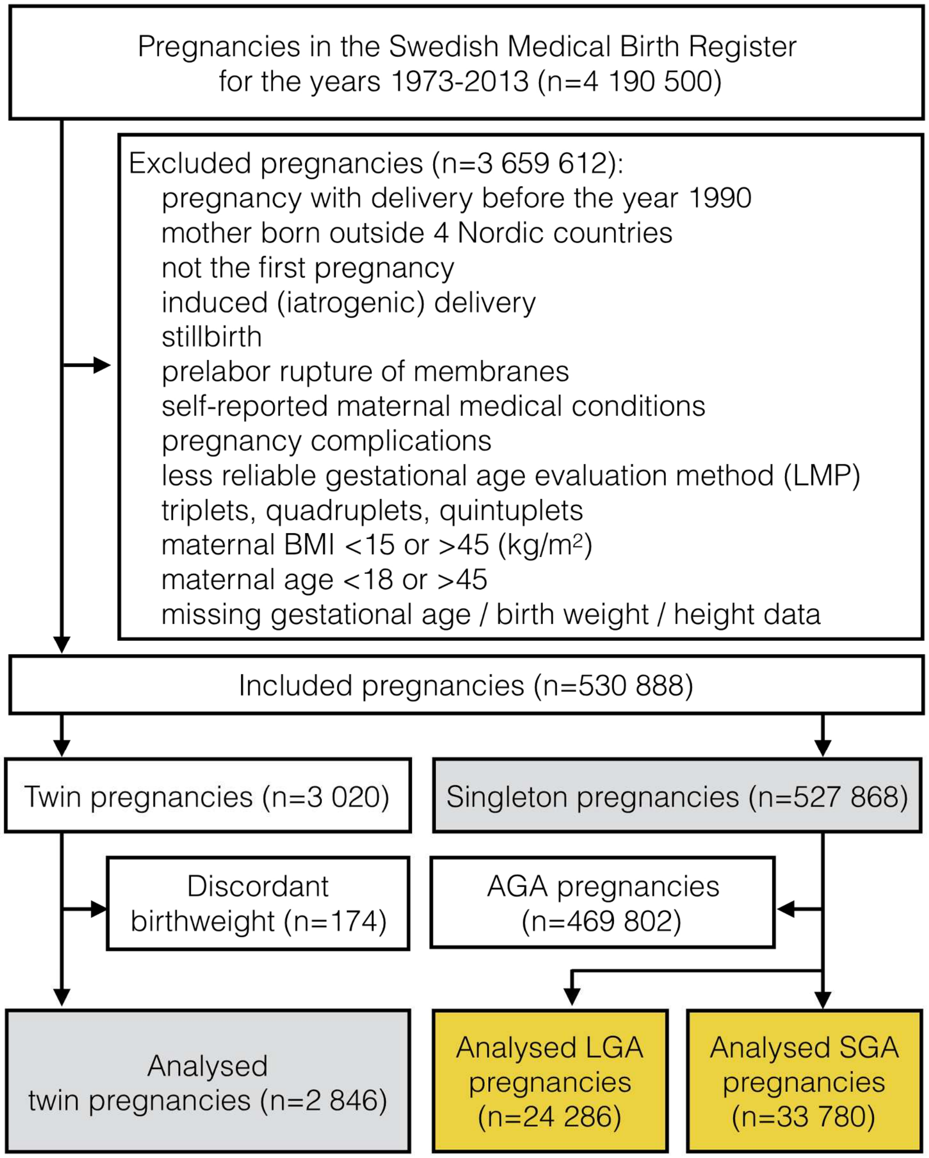

Figure 1. Flowchart for selection of analyzed pregnancies. AGA - child's birthweight is appropriate for gestational age; LGA and SGA - birthweight is large and small for gestational age, respectively. BMI - Body-mass index. LMP - last menstrual period. Two grey boxes represent the analysis in Model 1, two yellow boxes represent analysis in Model 2.

189x231mm (300 x 300 DPI)

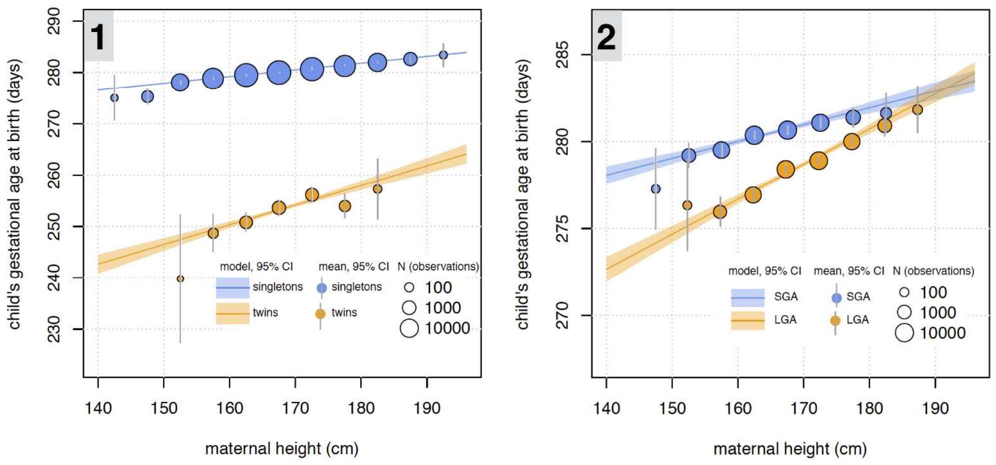

Figure 2. Panel 1) Interaction pattern in Model 1, where the uterine load is modeled as twin vs singleton pregnancies. **Panel 2)** Interaction pattern in Model 2, where uterine load is modeled as too large vs too small birthweight for gestational age in singleton pregnancies (LGA and SGA, respectively). Observations are grouped based on maternal height bins. CI - confidence interval. N - count of observations used to estimate mean. The interaction effect of maternal height and uterine load on gestational duration is demonstrated by the different slopes of the two regression lines calculated in the two groups with a different uterine load.

354x167mm (300 x 300 DPI)

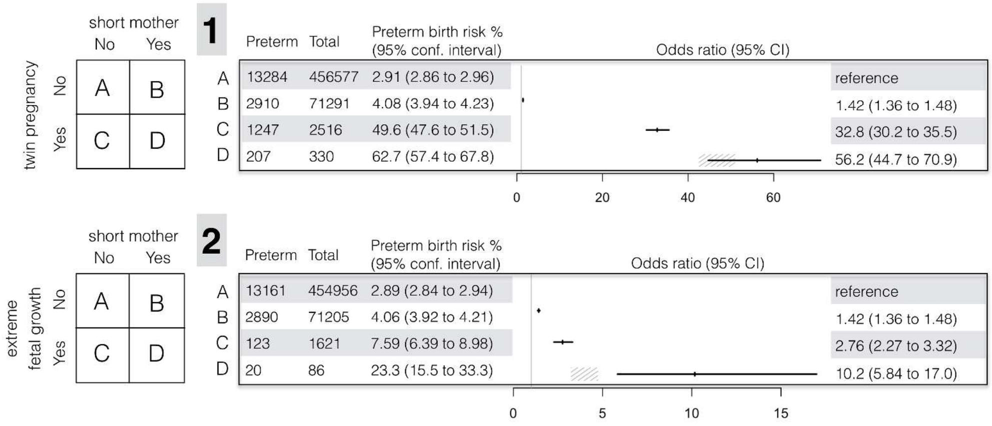

Figure 3. Preterm birth in short mothers with: **Panel 1)** twin pregnancies, **Panel 2)** extreme fetal growth. Short stature is defined as <161cm. Preterm birth is defined as delivery before 259 days of gestation. Extreme fetal growth is defined as >2.75 birthweight Z-score (adjusted for gestational age). The grey angular lines denote 95% confidence interval for the expected odds ratio in stratum D, estimated as the product of odds ratios in strata B and C with the assumption of no interaction. In Panel 1, the odds ratio in stratum D is larger than expected due to the interaction between maternal height and fetal growth (interaction P=0.1, logistic regression). In Panel 2, the odds ratio in stratum D is significantly larger than expected due to the interaction between maternal height and fetal growth (interaction P<0.0005, logistic regression).

330x141mm (300 x 300 DPI)

For peer review only

Supplementary File

"Uterine distention as a factor in birth timing: retrospective nationwide cohort study in Sweden". Bacelis J. et al. 2018.

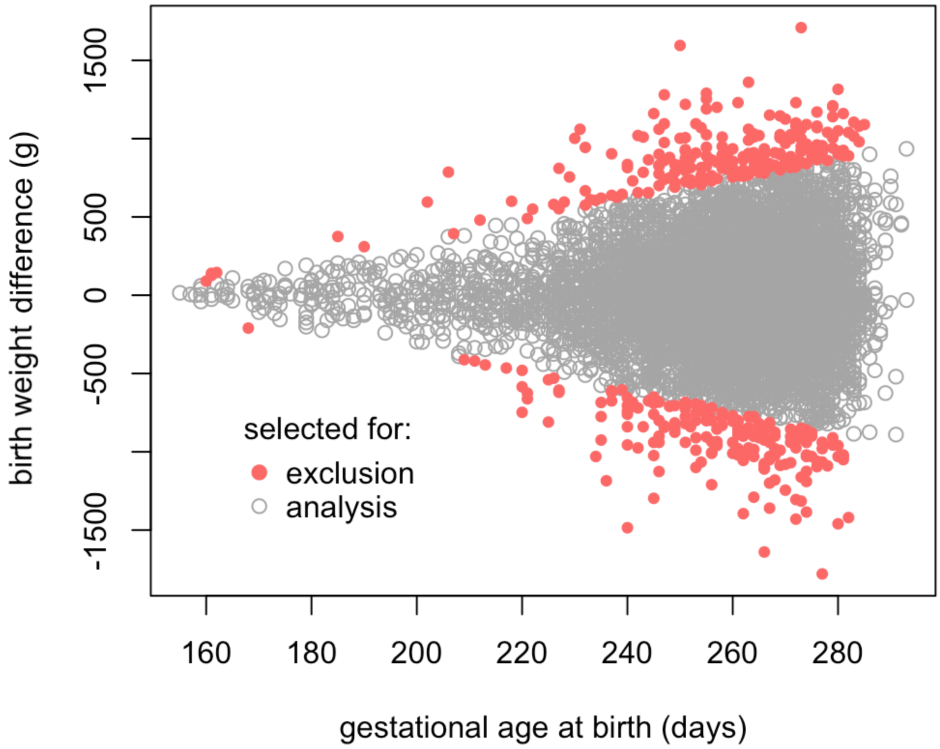

Sup Fig 1 I Selection of twin pregnancies. Pregnancies were excluded if birth weight of two twins differed by more than 2 SD of all intra-twin differences in that gestational age window. Pregnancies with thus discordant twin birth weights (n=174) were excluded from analyses.

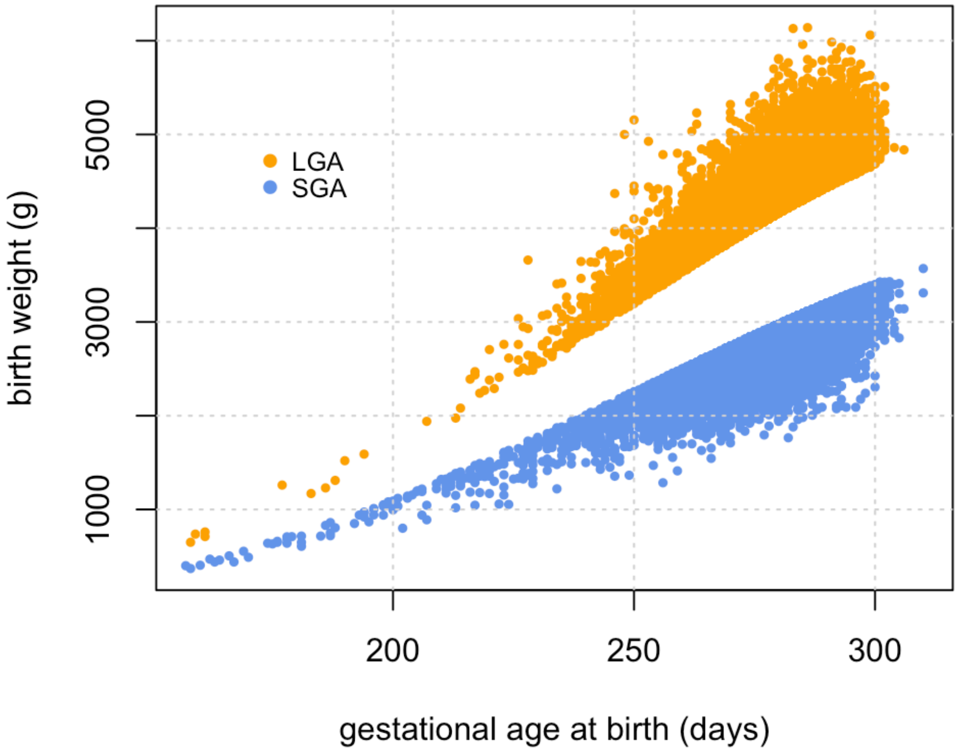

Sup Fig 2 I Classifying singleton births as small (SGA) or large (LGA) for gestational age using birth weight Z-score <-1.5 and >1.5, respectively. Birth weight Z-score was estimated from longitudinally derived intra-uterine growth curves in healthy term pregnancies using polynomial regression equation from Marsal et al.*****, which takes into account child's sex, birth weight, and ultrasound-determined gestational age at birth.

***** Marsal K, Persson PH, Larsen T, et al. Intrauterine growth curves based on ultrasonically estimated foetal weights. Acta Paediatr 1996;85(7):843-8. [published Online First: 1996/07/01].

Supplementary File

"Uterine distention as a factor in birth timing: retrospective nationwide cohort study in Sweden". Bacelis J. et al. 2018.

A

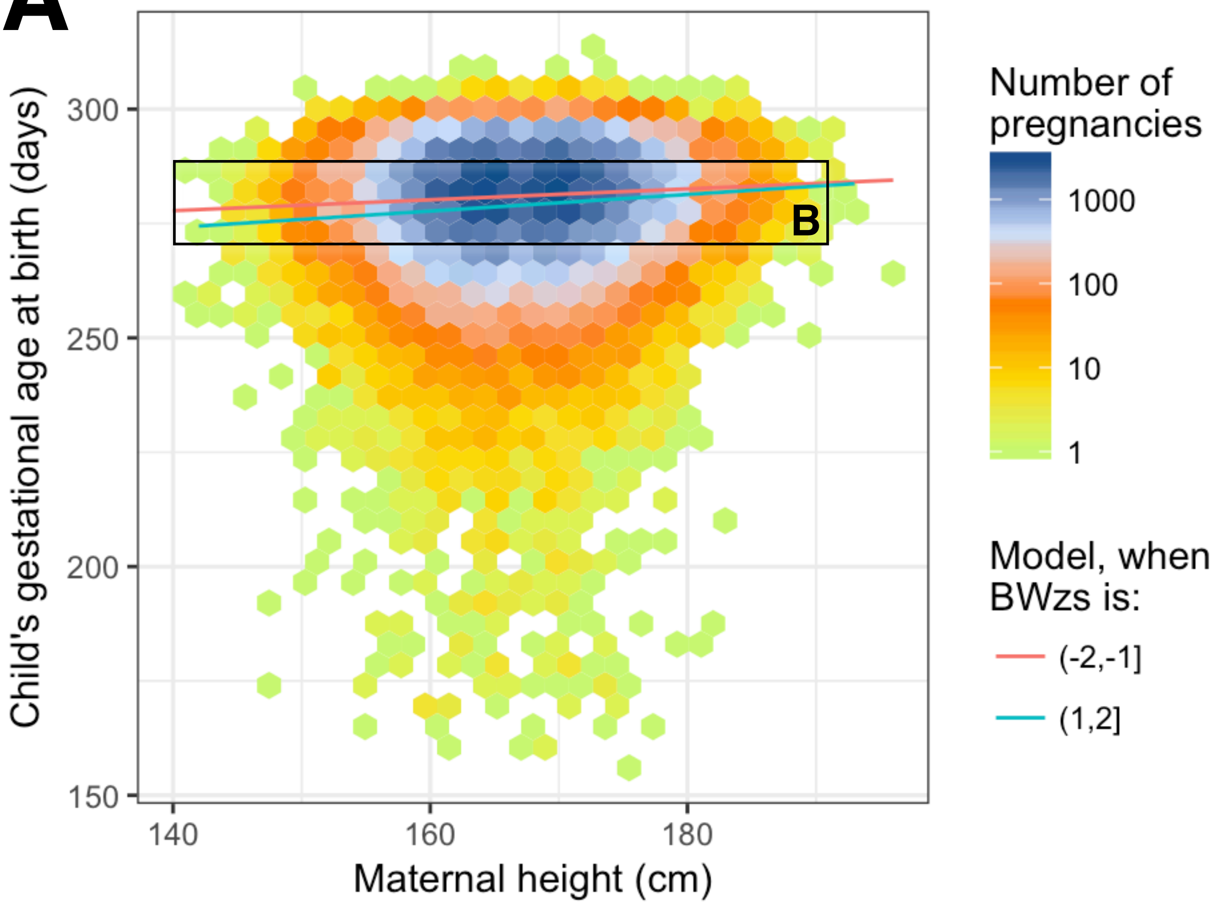

B

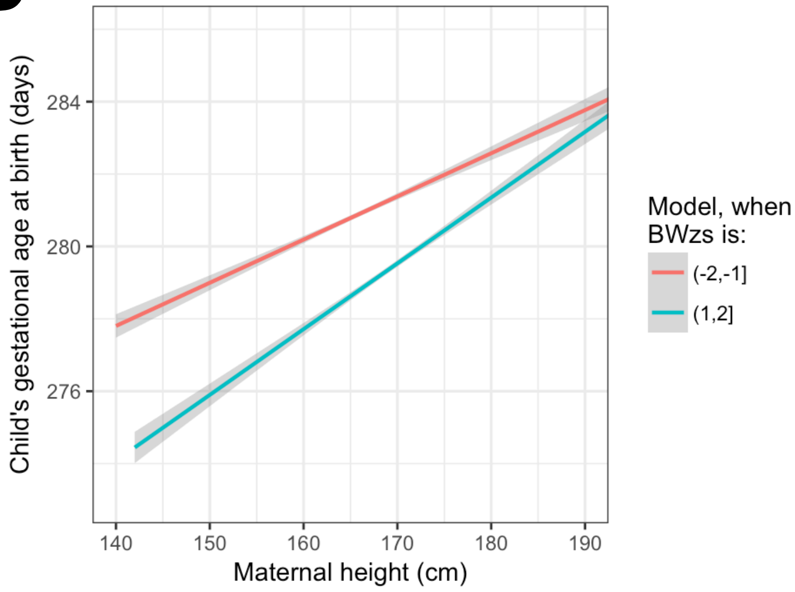

C

	Beta	Serr	Tval	Pval
**Intercept**	261,1	1,026	254,6	<1E-150
**MH**	0,119	0,006	19,3	9,4E-83
**LGA**	-12,46	1,69	-7,367	1,8E-13
**MHxLGA**	0,062	0,01	6,20	5,7E-10

Sup Fig 3 I Sensitivity analysis with different SGA and LGA definitions. SGA and LGA are defined by birth weight Z-score in ranges (-2.0, -1.0] and (1.0, 2.0], respectively. Total number of observations is 133 196 (SGA=82 471, LGA=50 725). Panel A: general view of interaction pattern; Panel B: zoomed-in view; Panel C: regression coefficients, where *MH* is maternal height and "*x*" indicates interaction. *BWzs* is birth weight Z-score.

For peer review only - http://bmjopen.bmj.com/site/about/guidelines.xhtml

Supplementary File

"Uterine distention as a factor in birth timing: retrospective nationwide cohort study in Sweden". Bacelis J. et al. 2018.

A

	Beta	SErr	Tval	Pval
**Intercept**	253,8	0,429	592	<1E-150
**MH**	0,157	0,003	61,3	<1E-150
**BWzs**	-4,80	0,491	-9,78	1.4E-22
**MHxBWzs**	0,024	0,003	8,28	1.3E-16

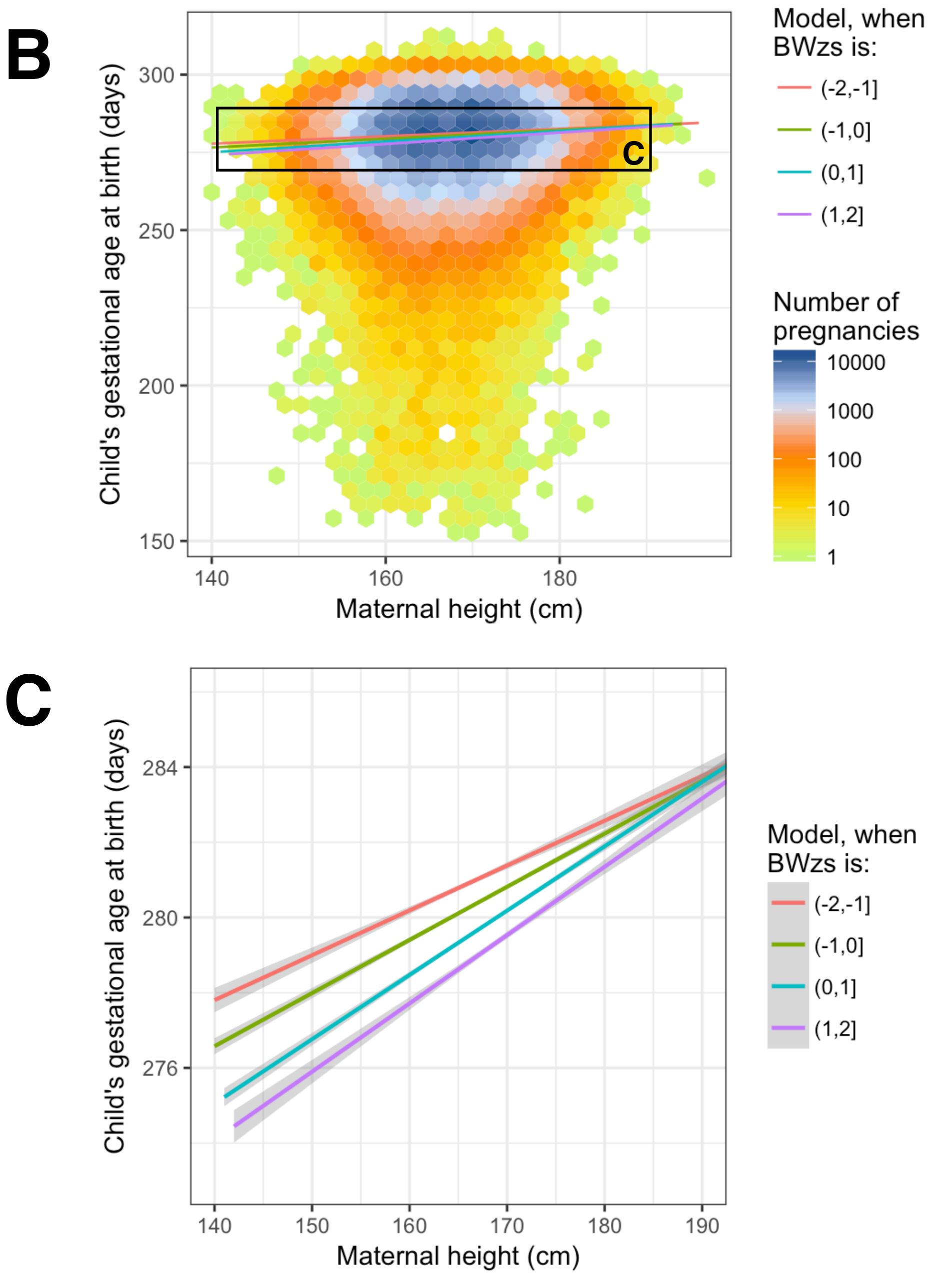

Sup Fig 4 I Sensitivity analysis with continuous uterine load variable. Only the appropriate for gestational age singletons were used, with birth weight Z-score in range (-2.0, 2.0]. In total, there were 508 442 observations. Panel A: results of regression analysis with interaction term; Panels B and C: intuitive aid for interpretation of regression coefficients (using categorized BWzs). *MH* is maternal height, *BWzs* is birth weight Z-score, and "x" indicates interaction.

Supplementary File

"Uterine distention as a factor in birth timing: retrospective nationwide cohort study in Sweden". Bacelis J. et al. 2018.

Sup Table 1 | Summary statistics of applied exclusions. This table supplements the flowchart in Figure 1, where it is equivalent to the block named "Excluded pregnancies".

order	exclusion-filter name	remaining rows	rows removed	fraction removed
1	maternal ID is missing	4185196	5304	0,13 %
2	duplicated maternal ID - child ID combination	4185184	12	0,0003 %
3	mother born (or national) outside of 4 Nordic countries	3625825	559359	13 %
4	missing, unreliable, <140 or >200 (cm) maternal height	2616038	1009787	27,8 %
5	maternal BMI <15 or >45 (kg/m^2)	2598666	17372	0,66 %
6	stillbirth	2587048	11618	0,45 %
7	missing gestational age at birth data	2584054	2994	0,12 %
8	less reliable gestational age evaluation method (LMP)	1834910	749144	29,0 %
9	induced (iatrogenic) delivery (or pregnancy before 1990)	1295490	539420	29,4 %
10	prelabor rupture of membranes (PROM)	1276244	19246	1,49 %
11	current Caesarean section	1273451	2793	0,22 %
12	previous Caesarean section	1206658	66793	5,25 %
13	self-reported maternal medical conditions	1188517	18141	1,50 %
14	pregnancy complications	1167804	20713	1,74 %
15	collapse rows to pregnancies (affects twins, triplets etc.)	1160063	7741	0,66 %
16	unclear twin/singleton assignment, triplets etc.	1159635	428	0,037 %
17	discordant maternal data in twin entries	1159542	93	0,008 %
18	birth weight is missing	1157760	1782	0,15 %
19	not the first pregnancy	535300	622460	53,8 %
20	maternal age <18 or >45	530888	4412	0,82 %

The authors would like to caution the reader in interpreting the "fraction removed" column: the applied filters overlap (i.e., are correlated), thus the fraction of removed pregnancies due to each filter is highly dependent on the order of applied filters and might not agree well with proportion of certain type of pregnancies estimated in other studies.

For peer review only - http://bmjopen.bmj.com/site/about/guidelines.xhtml

STROBE Statement—checklist of items that should be included in reports of observational studies

	Item No.	Recommendation	Page No.	Relevant text from manuscript
**Title and abstract**	1	(*a*) Indicate the study's design with a commonly used term in the title or the abstract	2	"Retrospective cohort study.."
		(*b*) Provide in the abstract an informative and balanced summary of what was done and what was found	2	"Statistical interaction between maternal height and uterine load categories.."
**Introduction**				
Background/rationale	2	Explain the scientific background and rationale for the investigation being reported	3	"..preterm delivery is the leading cause of childhood mortality.."
Objectives	3	State specific objectives, including any prespecified hypotheses	4	"..we hypothesed that birth is triggered by an interaction between uterine size and constantly increasing uterine load.."
**Methods**				
Study design	4	Present key elements of study design early in the paper	5	[pregnancies in Sweden of] "..mothers who were born in four Nordic countries: Sweden, Norway, Denmark and Finland."
Setting	5	Describe the setting, locations, and relevant dates, including periods of recruitment, exposure, follow-up, and data collection	5	"..Swedish Medical Birth Register.." "..1990 to 2013.." "..the first pregnancy of every mother was used.."
Participants	6	(*a*) *Cohort study*—Give the eligibility criteria, and the sources and methods of selection of participants. Describe methods of follow-up *Case-control study*—Give the eligibility criteria, and the sources and methods of case	5	"Deliveries initiated by elective caesarean section or starting with prelabor rupture of

For peer review only - http://bmjopen.bmj.com/site/about/guidelines.xhtml
For peer review only

		ascertainment and control selection. Give the rationale for the choice of cases and controls<br>*Cross-sectional study*—Give the eligibility criteria, and the sources and methods of selection of participants		membranes were removed. Further exclusions contained self-reported maternal medical conditions [...], pregnancy complications.."
		(*b*) *Cohort study*—For matched studies, give matching criteria and number of exposed and unexposed<br>*Case-control study*—For matched studies, give matching criteria and the number of controls per case		
Variables	7	Clearly define all outcomes, exposures, predictors, potential confounders, and effect modifiers. Give diagnostic criteria, if applicable	6<br><br>6	"..child's gestational age at birth (GA) being dependent on continuous maternal height (MH), uterine load (UL) categories.."<br>"..uterine load: [1] twin versus singleton pregnancies, and [2] LGA versus SGA pregnancies.."
Data sources/ measurement	8*	For each variable of interest, give sources of data and details of methods of assessment (measurement). Describe comparability of assessment methods if there is more than one group	*5,6*	"Maternal height.."<br>"Gestational age.."<br>"Twin pregnancies.."<br>"Fetal Growth.."
Bias	9	Describe any efforts to address potential sources of bias	6<br><br>7	"We used two [very different] models of uterine load.."<br>"..computer simulation [was used] to make sure that the used assumptions were sufficient.."
Study size	10	Explain how the study size was arrived at	5	Section "Study Population" and below

Continued on next page

For peer review only

For peer review only - http://bmjopen.bmj.com/site/about/guidelines.xhtml

Quantitative variables	11	Explain how quantitative variables were handled in the analyses. If applicable, describe which groupings were chosen and why	7	"..continuous maternal height (MH), uterine load (UL) categories.." and formula below
Statistical methods	12	(*a*) Describe all statistical methods, including those used to control for confounding	6<br>7	"multiple linear regression.."<br>"..logictic regression.."
		(*b*) Describe any methods used to examine subgroups and interactions	7	the whole page
		(*c*) Explain how missing data were addressed	Fig1	excluded pregnancies with "missing gestational age / birthweight / height data"
		(*d*) *Cohort study*—If applicable, explain how loss to follow-up was addressed<br>*Case-control study*—If applicable, explain how matching of cases and controls was addressed<br>*Cross-sectional study*—If applicable, describe analytical methods taking account of sampling strategy		not applicable
		(*e*) Describe any sensitivity analyses	12	"Sensitivity analyses showed.."
**Results**				
Participants	13*	(a) Report numbers of individuals at each stage of study—eg numbers potentially eligible, examined for eligibility, confirmed eligible, included in the study, completing follow-up, and analysed	Fig1	
		(b) Give reasons for non-participation at each stage	Fig1	
		(c) Consider use of a flow diagram	Fig1	
Descriptive data	14*	(a) Give characteristics of study participants (eg demographic, clinical, social) and information on exposures and potential confounders	5	"mothers who were born in four Nordic countries: Sweden, Norway, Denmark and Finland"<br>"Further exclusions contained.."
		(b) Indicate number of participants with missing data for each variable of interest	Fig1	[no individuals with missing data]
		(c) *Cohort study*—Summarise follow-up time (eg, average and total amount)		not aplicable
Outcome data	15*	*Cohort study*—Report numbers of outcome events or summary measures over time		not aplicable (our outcome is interaction three variables)
		Case-control study—Report numbers in each exposure category, or summary measures of exposure	Fig3	somewhat applicable
		Cross-sectional study—Report numbers of outcome events or summary measures		
Main results	16	(*a*) Give unadjusted estimates and, if applicable, confounder-adjusted estimates and their precision (eg, 95% confidence interval). Make clear which confounders were adjusted for and why they were		Not applicable. Confounder adjustment would be too

For peer review only

For peer review only - http://bmjopen.bmj.com/site/about/guidelines.xhtml

	included		complicated to interpret in this particular study investigating interaction effect. We used different approach (two different models of phenomenon and simulation) to address this problem.
	(*b*) Report category boundaries when continuous variables were categorized	6	"The newborns with a Z-score larger than 1.5 in the singleton cohort were classified as large for gestational age (LGA), and newborns with a Z-score lower than -1.5 in the singleton cohort were classified as small for gestational age (SGA).."
	(*c*) If relevant, consider translating estimates of relative risk into absolute risk for a meaningful time period		not relevant

Continued on next page

For peer review only - http://bmjopen.bmj.com/site/about/guidelines.xhtml

Other analyses	17	Report other analyses done—eg analyses of subgroups and interactions, and sensitivity analyses	12	"Sensitivity analyses showed.."
**Discussion**				
Key results	18	Summarise key results with reference to study objectives	12	"..the observed statistical interaction is likely a manifestation of biomechanical interplay between uterine size and uterine load (fetal size) in time.."
Limitations	19	Discuss limitations of the study, taking into account sources of potential bias or imprecision. Discuss both direction and magnitude of any potential bias	12<br>12<br>3	"..An alternative explanation.."<br>"..not when parity was higher.."<br>"..possible limitation of this study.."
Interpretation	20	Give a cautious overall interpretation of results considering objectives, limitations, multiplicity of analyses, results from similar studies, and other relevant evidence	13	"The observed interactions support the hypothesis .."
Generalisability	21	Discuss the generalisability (external validity) of the study results	12	"..not when parity was higher.."
**Other information**				
Funding	22	Give the source of funding and the role of the funders for the present study and, if applicable, for the original study on which the present article is based	13	"Funding.."

*Give information separately for cases and controls in case-control studies and, if applicable, for exposed and unexposed groups in cohort and cross-sectional studies.

Note: An Explanation and Elaboration article discusses each checklist item and gives methodological background and published examples of transparent reporting. The STROBE checklist is best used in conjunction with this article (freely available on the Web sites of PLoS Medicine at http://www.plosmedicine.org/, Annals of Internal Medicine at http://www.annals.org/, and Epidemiology at http://www.epidem.com/). Information on the STROBE Initiative is available at www.strobe-statement.org.

For peer review only

For peer review only - http://bmjopen.bmj.com/site/about/guidelines.xhtml
